# Force-Induced Ankle Opening Reveals Mechanical Stabilization of the Ankle of Human β‑Cardiac Myosin

**DOI:** 10.1021/acsnano.5c22720

**Published:** 2026-05-21

**Authors:** Surya Pratap S. Deopa, Kristen K. Bjorkman, Devin T. Edwards, Marc-André LeBlanc, Massimo Buvoli, Steven H. Oakes, Brandon Dzuba, Anastasia Karabina, Leslie A. Leinwand, Thomas T. Perkins

**Affiliations:** † JILA, National Institute of Standards and Technology and University of Colorado, Boulder, Colorado 80309, United States; ‡ Department of Molecular, Cellular, and Developmental Biology, University of Colorado, Boulder, Colorado 80309, United States; § BioFrontiers Institute, University of Colorado, Boulder, Colorado 80303, United States; ∥ Department of Physics, University of Colorado, Boulder, Colorado 80309, United States

**Keywords:** atomic force microscopy, myosin, single-molecule force spectroscopy, regulatory light chain, hypertrophic cardiomyopathy, coarse-grained simulation

## Abstract

Human β-cardiac myosin drives contraction in the heart. Extensive biophysical and single-molecule studies have quantified myosin’s chemo-mechanical cycle, which generates ∼5 nm of displacement and 5–7 pN of force. Myosin’s 9 nm-long, α-helical lever arm is rigidified by bound essential and regulatory light chains (ELC and RLC). Numerous pathogenic mutations and sequence-conservation patterns within the lever arm where the RLC binds (LA^RLC^) belie the overly simplified view that the lever arm acts solely as a rigid rod that transduces ATP hydrolysis into motion. Structural studies have shown that myosin adopts an interacting-heads motif (IHM), which inhibits motor activity and mechanically strains the RLC complex, consisting of the RLC bound to the LA^RLC^. Alteration in the configuration of the RLC complex’s “ankle”—a sharp kink in the lever arm—is hypothesized to modulate the propensity of myosin to enter the IHM. To investigate the complex’s mechanical stability, we developed a single-molecule atomic-force microscopy assay with three different pulling geometries: pulling across the LA^RLC^, the RLC, and the RLC complex. When pulling across the LA^RLC^ by applying force to its N and C termini, the mechanical dissociation of the RLC was resolved along with two intermediates. Coarse-grained Brownian dynamics detailed these molecular configurations as the opening of myosin’s ankle and the preferential dissociation of one of the RLC’s two EF-hand domains. Moreover, the linker between the EF-hand domains forms an interface with an RLC N-terminal loop. This interface stabilized the native acute ankle angle against opening. Pulling across the RLC and the RLC complex revealed different unfolding pathways, each with one intermediate. Looking forward, these assays can probe for the effects of pathogenic mutations and phosphorylation on the nanomechanics of the RLC complex.

## Graphical abstract

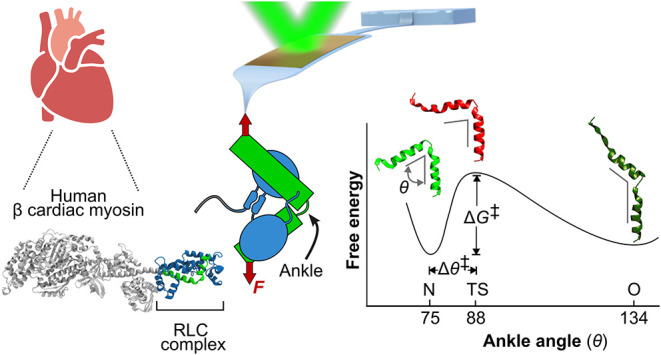

Human β-cardiac myosin is the primary molecular motor that drives heart contraction. Mutations in human β-cardiac myosin and other sarcomeric proteins are a major cause of hypertrophic cardiomyopathy (HCM),^1^ which affects up to 1 in 500 adults and is the leading cause of sudden death among young adults.^2,3^ However, an understanding of the underlying molecular mechanisms of most myosin mutations that cause HCM remains elusive.^4^ Additionally, the majority of studies have concentrated on the enzymatic motor domain of myosin, despite disease-causing mutations mapping to all structural and functional domains of the molecule.^5^ This lack of experimental attention includes myosin’s lever arm, a 9 nm-long α helix rigidified by the binding of the essential and regulatory light chains (ELC and RLC) (Figure 1A).^6^ The lever arm amplifies the ATP-driven conformational rotation into nanometer-scale motion.^7^ It also contains a sharp bend, termed the “hook” or the “ankle”,^8^ within the lever arm where the RLC binds (LA^RLC^) to form the RLC complex (Figure 1B). Notably, an unusually high number of HCM-causing mutations map to the LA^RLC^: 31 at 21 sites within the 37-amino-acid-long lever-arm segment (Table S1, Figure S1).^5^ This frequency of disease-causing mutations is 2.3 times higher than the average mutation frequency across human β-cardiac myosin^5^ and suggests a critical, yet incompletely described role for the LA^RLC^ and, more generally, the RLC complex.

**Figure 1:**
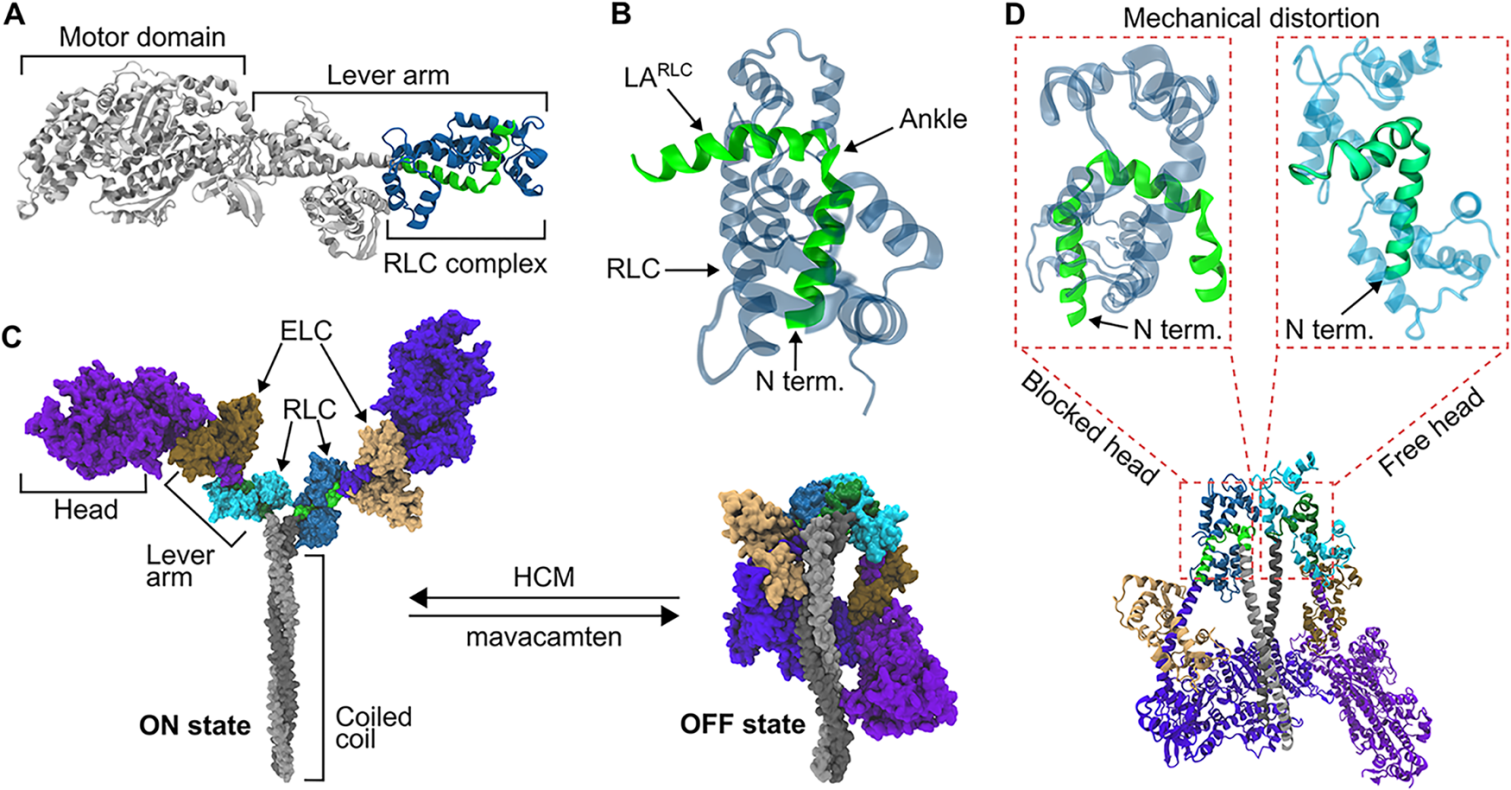
Regulatory light chain (RLC) complex of human β-cardiac myosin. (A) Ribbon diagram of a homology model of the myosin S1 fragment,^13^ with the RLC and the cognate segment of the lever arm where the RLC binds (LA^RLC^) highlighted in blue and green, respectively. (B) Structure of the isolated RLC complex, as predicted by AlphaFold 3,^56^ where the break in the lever-arm α helix is the ankle (or hook). (C) Schematic denoting the equilibrium between myosin’s ON and OFF states, structurally represented using homology models,^13,14^ that modulates the availability of myosin to generate force in the context of the sarcomere. Several hypertrophic cardiomyopathy (HCM) causing mutations shift the equilibrium toward the ON state, while mavacamten shifts the equilibrium to the OFF state.^19,20^ (D) Cyro-EM resolved structure of the OFF state shows the lever arm hinges around the RLC complex to fold back onto the coiled coil (PDB: 8ACT^17^). (Inset) RLC complexes in the OFF state show mechanical distortion at and immediately adjacent to the LA^RLC^, where two RLC complexes experience different strains due to the asymmetric nature of the OFF state for the “blocked” and “free” head.

In the traditional swinging lever-arm hypothesis, the lever arm functions solely as a rigid rod that transduces ATP hydrolysis into motion.^7^ Structurally, this hypothesis is supported by the crystal structure of myosin that includes the 9-nm lever arm.^6^ Functionally, Uyeda et al. demonstrated that the sliding velocity scaled linearly with lever-arm length in an *in vitro* motility assay, directly supporting the lever-arm hypothesis.^9^ Lever-arm length dictating step size is further supported by the larger step size of myosin V with its longer lever arm.^10^ This view is reinforced by a sequence alignment among 9 paralogs of class II human myosins, which shows high sequence diversification within the 61-amino-acid (aa) lever arm (43% sequence identity) (Table S2A). This analysis implies that a variety of protein sequences could adopt the structurally required α helix. However, this sequence diversity is not due to relaxed selective pressure: a sequence alignment of β-cardiac myosin orthologs from 10 mammalian species revealed very high sequence conservation^11^ (93% identity, 97% similarity; Table S2B). This comparison of myosin paralogs within species and orthologs across species coupled with the numerous pathogenic mutations supports a functional specialization of the LA^RLC^ component of the RLC complex beyond the simple swinging lever-arm hypothesis.

Indeed, existing work suggests that the nanomechanics of the RLC complex play a role in myosin’s function. In scallop smooth muscle myosin, the ankle adopts two different angles that are hypothesized to add crossbridge compliance,^12^ a critical parameter in muscle physiology. More recent work in striated muscle myosin has hypothesized that the ankle configuration of the RLC complex may play an important role in regulating the activity of myosin in the context of the sarcomere by changing the partitioning^11^ of myosin between catalytically active^13^ and inhibited states^14^ (Figure 1C). In the OFF state, myosin adopts a pretzel-like configuration—referred to as the interacting-heads motif (IHM)—where the heads are sequestered away from binding actin.^15–17^ We note that in smooth muscle myosin, phosphorylation of RLC’s N-terminal tail acts as an on/off switch that destabilizes the OFF state, while, in cardiac myosin, such phosphorylation is not strictly required for adoption of the ON state, but its effect is weaker and more modulatory.^17^ Biochemically, such sequestration reduces myosin’s actin-activated hydrolysis of ATP.^18^ The nanomechanics of the RLC complex is potentially important because the RLC complex is under enhanced strain in the OFF state, particularly near the C-terminal end of the LA^RLC^ for both the blocked and free head (Figure 1D). Indeed, the underlying mechanism for an ankle adjacent, HCM-causing mutation (F834L) is hypothesized to be a change in ankle angle orientation within the RLC complex.^11^ This change, in turn, would bias myosin to adopt the ON state, leading to hypercontractility and, therefore, the observed HCM. Reinforcing this strain-dependent activity hypothesis, the HCM drug mavacamten (Camzyos), works by stabilizing the OFF state, decreasing the pathological hypercontractility (Figure 1C).^19,20^

While these multiple lines of evidence highlight the functional and biomedical importance of the RLC complex, we hypothesized that the nanomechanics of the RLC complex could affect muscle physiology by multiple mechanisms, including modulating crossbridge compliance^12^ and/or changing the partitioning between the ON and OFF states due to myosin’s ankle-angle orientation.^17^ Yet, a more complete nanomechanical characterization of the RLC complex has remained elusive. The three-bead optical-trapping assay^21^ primarily probes myosin activity within the context of the lever-arm hypothesis, yielding key parameters such as step size, force, and strongly bound state lifetime.^21–24^ Here, we characterize the nanomechanics of the RLC complex by introducing a single-molecule atomic-force microscopy (AFM) assay to probe the stability and dynamics of the RLC complex in three different pulling geometries: (1) pulling across the LA^RLC^ by applying force to its N and C termini, (2) pulling across the RLC (a calmodulin-like domain) by applying force to its N and C termini, and (3) pulling across the RLC complex by applying force to the N-terminus of the RLC and the C-terminus of the LA^RLC^ (Figure 2A–C). When pulling across the LA^RLC^—our best proxy for ankle stiffness—we resolved ankle opening as a discrete transition against an applied load. This result makes the human β-cardiac myosin RLC complex distinct from the ankle flexibility observed in scallop smooth muscle myosin inferred from its crystallizing into two structures with distinct ankle angles in the same unit cell.^12^ Coarse-grained Brownian dynamics detailed the molecular configurations of the RLC complex in its native acute ankle angle state (75°) and at an average open ankle angle of 140° under force (∼30 pN). And, when combined with dynamic force spectroscopy,^25,26^ these simulations enabled deduction of the angular rotation to the transition state (Δθ^‡^ = 13°). A force-propagation analysis^27^ showed that the native state’s acute ankle angle was stabilized by a loop–loop interaction between a small loop in the RLC’s N-terminal tail immediately before the start of helix A (residue 23) and the linker connecting the EF-hand domains (Figure 2A). When the N-terminal tail of the RLC was deleted, the ankle-opening force decreased, experimentally confirming the structural importance of this computationally predicted loop–loop interaction. Thus, for human β-cardiac myosin, this work establishes the presence of a significant energetic barrier to a new open ankle state of myosin’s lever arm and, more generally, describes a set of mechanical “fingerprints” for the RLC complex that forms the foundation for future studies probing the effects of pathogenic mutations.

**Figure 2:**
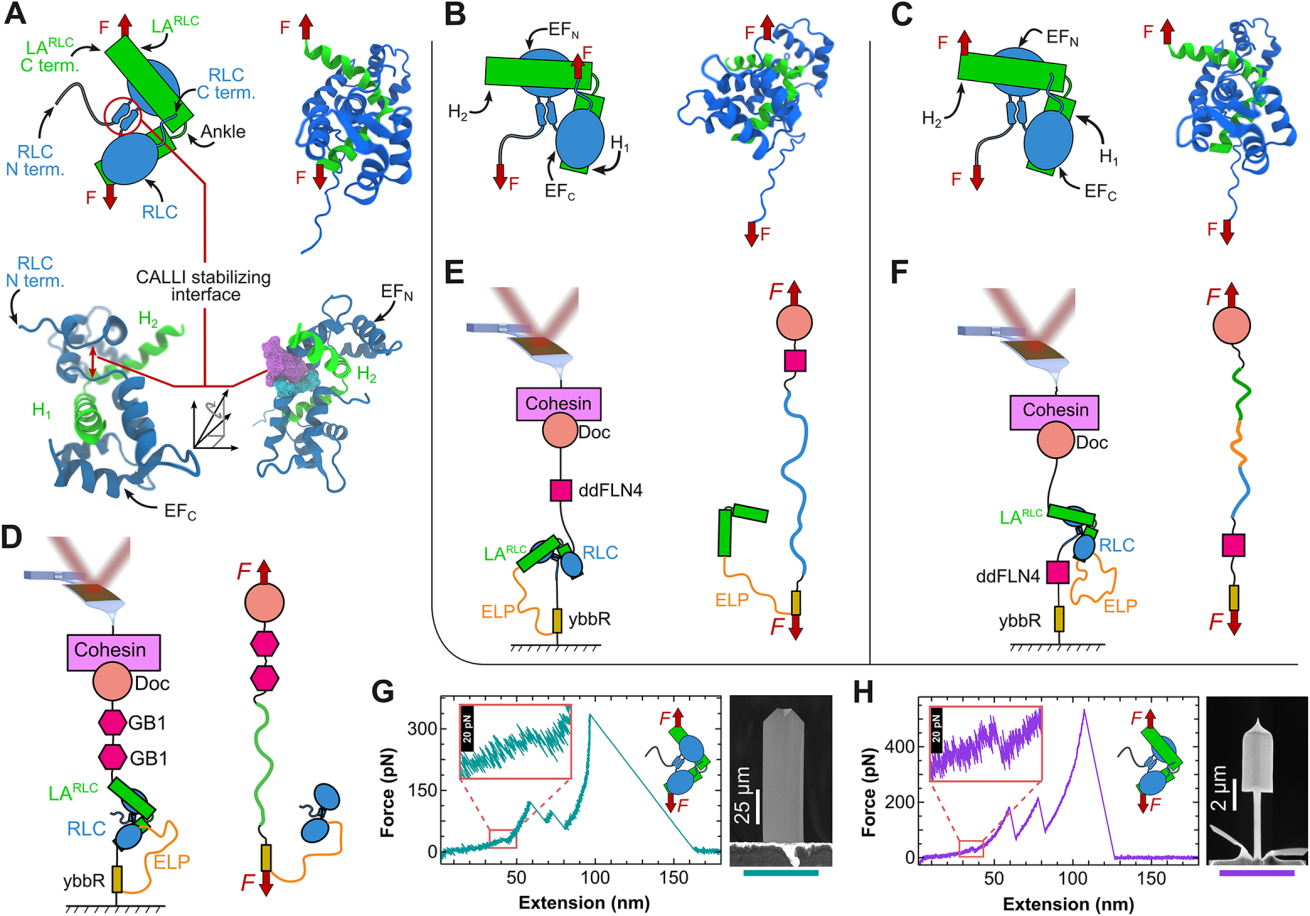
A single-molecule assay for probing the nanomechanics of the RLC complex. (A–C) Structural illustrations and accompanying ribbon diagrams of the three pulling geometries: pulling across the LA^RLC^ by applying force to its N and C termini, pulling across the RLC by applying force to its N and C termini, and pulling across the RLC complex by applying force to the N-terminus of the RLC and C-terminus of the LA^RLC^, respectively. Force is applied at the locations indicated by the red arrows. The ankle separates the lever-arm α helix into two parts, denoted helix 1 (H_1_) and helix 2 (H_2_). The RLC, a calmodulin-like fold, is composed of an N-terminal and C-terminal EF-hand domain (EF_N_ and EF_C_). The cardiac ankle loop–loop interface (CALLI) between a small loop in the RLC’s N-terminal tail and a loop that connects the RLC’s two EF-hand domains is depicted with a ribbon diagram (red arrow, left) and with the complementary molecular surfaces of a slightly larger region [Met^20^-Gln^25^ (purple) and Leu^90^–Pro^95^ (cyan), right] where the two ribbon diagrams are related by a 93° rotation, as indicated. (D) A schematic of the AFM-based force-spectroscopy assay shows the polyprotein used to apply force across the LA^RLC^ in a folded and unfolded state. Starting from the N-terminus, it consists of the RLC (blue), a 120-nm elastin-like peptide^30^ (ELP, orange), ybbR (gold), the LA^RLC^ (green), two copies of GB1 (a well characterized marker protein, red), and dockerin (peach). The internal ybbR tag was used to site-specifically anchor the polyprotein to a PEG-coated surface and thereby apply a force across the LA^RLC^ while leaving the RLC under essentially no tension. Cohesin (magenta) was covalently linked to a PEG-coated AFM cantilever and enables pulling on the polyprotein via the mechanically robust but reversible cohesin-dockerin interaction.^27^ (E, F) Schematic of assay and the polyproteins used to apply force across the RLC and across the RLC complex in both the folded and unfolded state. Note, the use of ddFLN4 as a marker protein. ddFLN4, which unfolds at lower force than GB1, is not preferred when pulling across the LA^RLC^, due to the high force needed to fully dissociate the RLC in that geometry. (G, H) For pulling across the LA^RLC^, representative force-extension curves at a constant velocity (600 nm/s) using a traditional length cantilever (*L* = 100 μm, panel G) stripped of gold and chrome for improved force stability^36^ and a focused-ion-beam (FIB) modified ultrashort cantilever (*L* = 10 μm, panel H) show the traditional length cantilever only marginally detects a mechanical transition when pulling across the LA^RLC^, while this transition was clearly resolved with the FIB-modified ultrashort cantilever. Data smoothed to 25 kHz. SEM images highlight the difference in size and geometry between the two AFM cantilevers.

## Results and Discussion

### A High-Resolution Force-Spectroscopy Assay for Unfolding the Lever Arm Bound by the RLC

To isolate the application of force to just across the LA^RLC^, we developed a polyprotein composed of the RLC and LA^RLC^ with integrated site-specific handles, ybbR^28^ and dockerin (Figure 2D).^29^ The polyprotein was designed with the RLC at the N-terminus followed by an elastin-like polypeptide^30^ (ELP) to provide enough flexibility for the RLC to bind to the internal LA^RLC^. The ELP was followed by ybbR, a small peptide tag^28^ that mechanically attached the polyprotein to the surface. The polyprotein continued with the LA^RLC^, two copies of GB1, a well characterized marker protein used to screen for single-molecule attachments,^31^ and finally dockerin. Note, we used GB1 as a marker protein when pulling across the LA^RLC^ because GB1 unfolds at higher force than the marker protein ddFLN4 ^32^ used in our other two polyproteins (Figure 2E,F). This change effectively minimized the probability of the marker protein unfolding before the mechanical dissociation of the RLC from the LA^RLC^ due to the unexpectedly high force needed to dissociate it (see below). Tethering of the RLC to the LA^RLC^ within the same polyprotein was critical to avoid aggregation, a common problem with bare α-helices.^33^ Moreover, by using a 120 nm-long ELP, we decreased the internal tension to an effectively negligible force (∼1 pN), an issue since the ELP is stretched in parallel as the AFM tip pulls the LA^RLC^ away from the surface during retraction (Figure 2D, see Methods for additional details). We note that the 37-aa LA^RLC^ started at Ser^810^ and went through Glu^846^ (Table S3), where Glu^844^–Glu^846^ were added due to substoichiometric binding of the RLC to human β-cardiac myosin S1 ending in the absence of those added amino acids (Ruppel, K.M. and Spudich, J.A. Stanford University, Stanford, CA, Personal communication, 2025).

The polyprotein was covalently anchored to a PEG-coated coverslip and attached to a PEG-coated AFM tip via a strong but reversible interaction. PEG-coating minimizes nonspecific tip–sample adhesion, which facilitates studying complexes that unfold at low forces by AFM standards.^34,35^ Specifically, the polyprotein was site-specifically anchored via ybbR to a Coenzyme A (CoA)-derivatized coverslip (Figure 2D).^29^ The C-terminal element in the polyprotein, dockerin, attached to a cohesin-labeled AFM tip via an exceptionally strong protein–protein interaction that ruptures at high forces (>400 pN).^29^ In this geometry, the polyprotein to the N-terminal side of the ybbR tag—including the RLC— experienced effectively no force during pulling.

We initiated the AFM-based pulling assay by gently (∼40–80 pN) pressing the cohesin-labeled cantilever into the polyprotein-labeled surface for a brief period (∼0.1 s) and then retracting it at a fixed velocity (*v*). The resulting force-extension curve clearly showed the unfolding of the two GB1 domains followed by the high force rupture of the cohesin-dockerin interaction (Figure 2G). But, the mechanical dissociation of the RLC was only marginally detected when using a standard length AFM cantilever [Olympus BioLever Long (*L* = 100 μm; *k* = 5 pN/nm)], despite being stripped of its gold and underlying chrome coating for improved force stability.^36^ This result was not unexpected because (1) the structured part of the LA^RLC^ corresponds to just 30 aa; (2) the LA^RLC^ is already in an extended α-helical conformation and therefore its expected unfolding signal would be smaller than for a compact folded domain; and (3) the noise floor of traditional AFM studies is ∼10 pN.^37^

To improve resolution, we switched to focused-ion-beam (FIB)-modified ultrashort cantilevers^38^ (*L* = 10 μm; *k* ≈ 15–25 pN/nm)] in a war-hammer geometry.^39^ These cantilevers provided time resolution of 2 μs coupled with sub-pN force precision over a broad time range (∼0.0004–30 s) (Figure S2A). In comparison to an uncoated BioLever Long, there was a ∼80-fold improvement in time resolution (Figure S2B).^40^ With these cantilevers, we resolved a clear mechanical fingerprint when pulling across the LA^RLC^ at 600 nm/s (Figure 2H). We note that this pulling speed is on par with the 400 nm/s estimate for the contraction velocity of a half sarcomere.^41^

### Mechanical Dissociation of the RLC Occurs via Two Intermediates

FIB-modified ultrashort cantilevers have previously resolved a multitude of hidden metastable intermediate states in the unfolding pathway of a membrane protein.^42,43^ Here, we resolved two such intermediates when pulling on an extended α-helical structure stabilized by the binding of the RLC (Figure 3A, red asterisk), where a state is generally defined as a continuous segment of a force-extension curve well described by a worm-like-chain (WLC) model^44,45^ separated by discrete force drops. This definition is modified slightly for the second intermediate, as detailed below. At a pulling speed of 600 nm/s, the first intermediate was not always observed (Figure S3A). The probability of observing 1 or 2 intermediates was 25% and 75% (*N* = 100), respectively. The force required to exit the starting, native state at this pulling velocity was 30.0 ± 0.6 pN (mean ± SEM; *N* = 100 @ ∼1100 pN/s), a high force given that all α-helical proteins tend to unfold at lower forces.^46–48^ Complete mechanical dissociation of the RLC from the second intermediate did not always proceed via an abrupt transition, rather there typically was a gradual transition over a broad force range (∼50–140 pN) to a fully unfolded state (Figure S3B–D). The origin of this behavior is discussed below. For the 15% of records showing a discrete transition to the fully unfolded state, this transition occurred at 110 ± 9 pN (mean ± SEM; *N* = 15).

**Figure 3:**
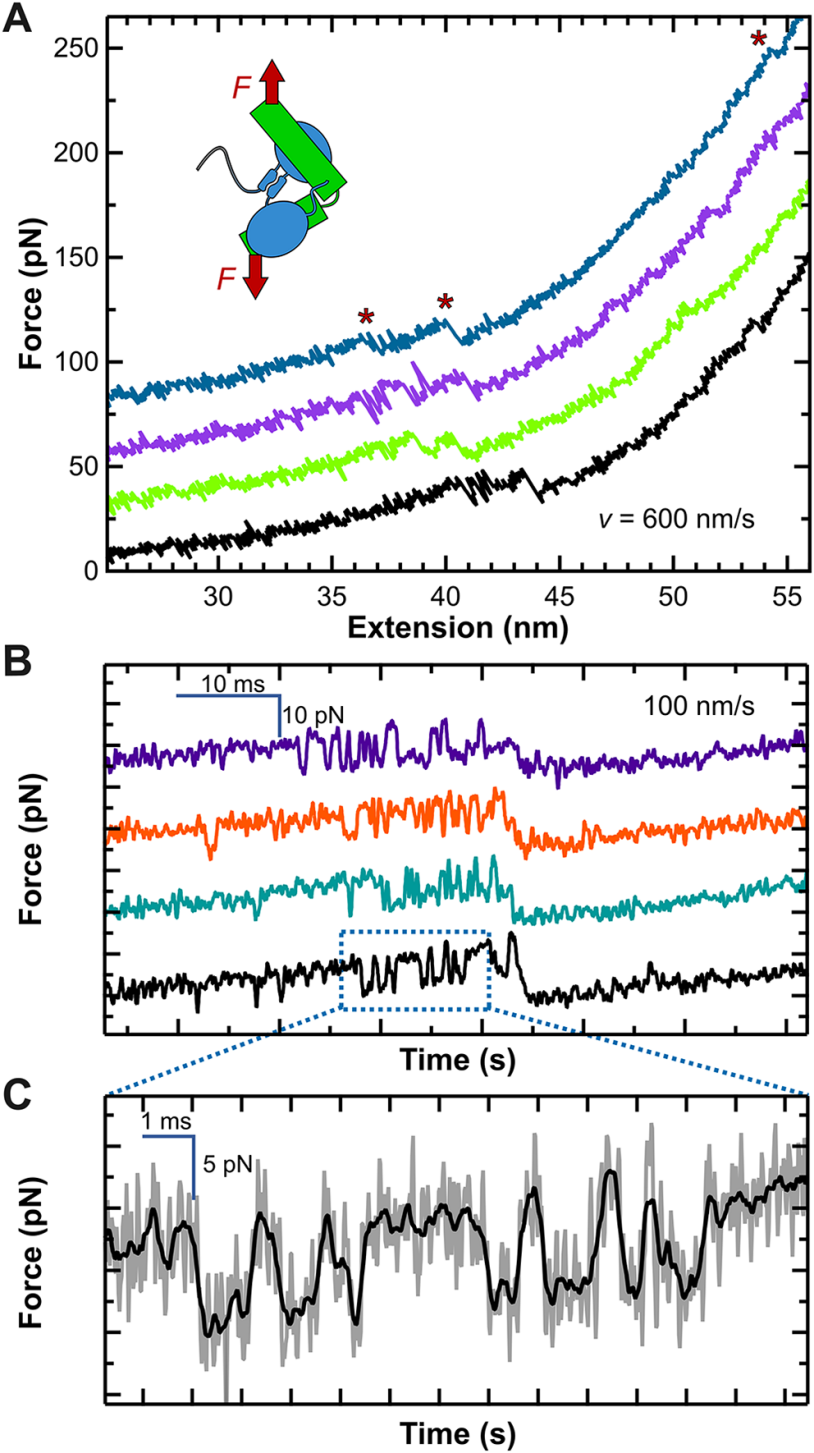
Two intermediates revealed when pulling across the LA^RLC^. (A) Force-extension curves showing three transitions (denoted with *) when pulling at *v* = 600 nm/s. Colored traces offset vertically for clarity and data smoothed to 2.5 kHz. (B) Force-vs-time traces show near equilibrium dynamics between the native and first intermediate when pulling at 100 nm/s. Data smoothed to 500 Hz. (C) Short lived (<1 ms), back-and-forth transitions between the native state and the first intermediate. Data smoothed to 500 Hz and 25 kHz (black and gray, respectively).

### Near-Equilibrium Transitions during Slower Pulling

Historically, AFM assays have tended to pull relatively fast due to limited force precision and stability.^37^ To investigate unfolding at closer to static loads, we retracted the cantilever more slowly (100 nm/s). With the enhanced performance of our FIB-modified cantilevers (Figure S2), we resolved multiple back-and-forth-transitions between the native state and the first intermediate (Figure 3B). Indeed, short-lived states (<1 ms) separated by just ∼ 5 pN were well resolved in high bandwidth data (Figure 3C, gray).

### Molecular Configuration of Intermediates Revealed by Coarse-Grained Simulations

The AFM data alone could not ascribe the mechanical intermediates to specific molecular conformations. So, we performed coarse-grained simulations to gain structural insight into the observed intermediates. Besides coarse-grained Brownian dynamics simulations being computationally much less intensive than all-atom simulations, they enable much longer run times (860 μs here) with simulated pulling speeds (40 μm/s) that are relatively close to those used in the experiments (0.1–10 μm/s). Notably, with these long run times, our simulations showed near-equilibrium conformational dynamics, as shown below. In contrast, a typical pulling velocity for all-atom steered molecular dynamics is 2.5 Å/ns (0.25 m/s),^27^ 4–6 orders of magnitude faster than typical AFM pulling speeds. Such simulations are too short (typically ∼1 μs) for back-and-forth conformational dynamics to occur. We note that coarse-grained molecular dynamics^49^ offer a middle ground of pulling speeds between all-atom simulations and coarse-grained Brownian dynamics.

Coarse-grained Brownian dynamics simulations have been quite useful in interpreting single-molecule force spectroscopy studies.^50–54^ We used the SOP-SC model from Liu et al.^55^ that parametrizes each amino acid as two interaction centers, one corresponding to the location of the C_α_ carbon and the second located at the center of mass of the side chain. To make efficient use of GPU acceleration, we simulated nine molecules in parallel with a base time step of 0.215 ps at a pulling velocity of 40 μm/s for a total of 27 simulations (Movie S1).

With no high-resolution structure of the human β-cardiac myosin RLC complex as the starting point for the simulation, we generated one using AlphaFold 3,^56^ as shown in Figure 1B, using the top-ranked structure out of 40 (see Methods for details). Importantly, AlphaFold 3 predicted with confidence the Cardiac Ankle Loop-Loop Interface (CALLI) between the linker connecting the RLC’s two EF-hand domains (Leu^90^–Pro^95^) and a small 3-aa loop (Met^20^–Glu^22^) before helix A of the RLC structure (Figure 2A, bottom left panel). CALLI, as will be shown below, helps stabilize the acute ankle angle against opening. We define CALLI to include a slightly larger region up to Gln^25^. The complementary nature of these molecular surfaces across this loop–loop interface is illustrated in Figure 2A (bottom right panel).

Importantly, AlphaFold 3 evaluated its prediction of the location of all these residues forming CALLI as “confident” to “very high” [pLDDT ≥ 70 (blue and dark blue, respectively), where the color code represents the degree of confidence in the residue location (Figure 4A)], with 70% of residues predicted with very high confidence. AlphaFold 3 goes on to predict an immediately adjacent short, 3-aa N-terminal tail α helix with confidence. We next compared the structure used as the basis for the simulation, with six similarly ranked predicted structures (see Methods for details). The superimposed structures were essentially indistinguishable for pLDDT ≥ 70 (Figure S4). The structurally unresolved, flexible N-terminal tail of the RLC (residues 1–19) in the cryoEM structure of the human β-cardiac IHM^17^ and crystallographic studies of the homologous RLC complex^12^ leads to the primary source of variability (pLDDT ≤ 70) along with the last 5 aa at the C-terminal end of LA^RLC^. More quantitatively, the average backbone RMSD between these six other structures for pLDDT ≥ 70 and the structure used as the basis for simulation was a mere 0.19 Å and the prediction of CALLI was even more precise (0.096 Å), providing confidence that AlphaFold 3 predicted a unique, well-constrained structure of the RLC complex as the starting basis for the simulations.

**Figure 4:**
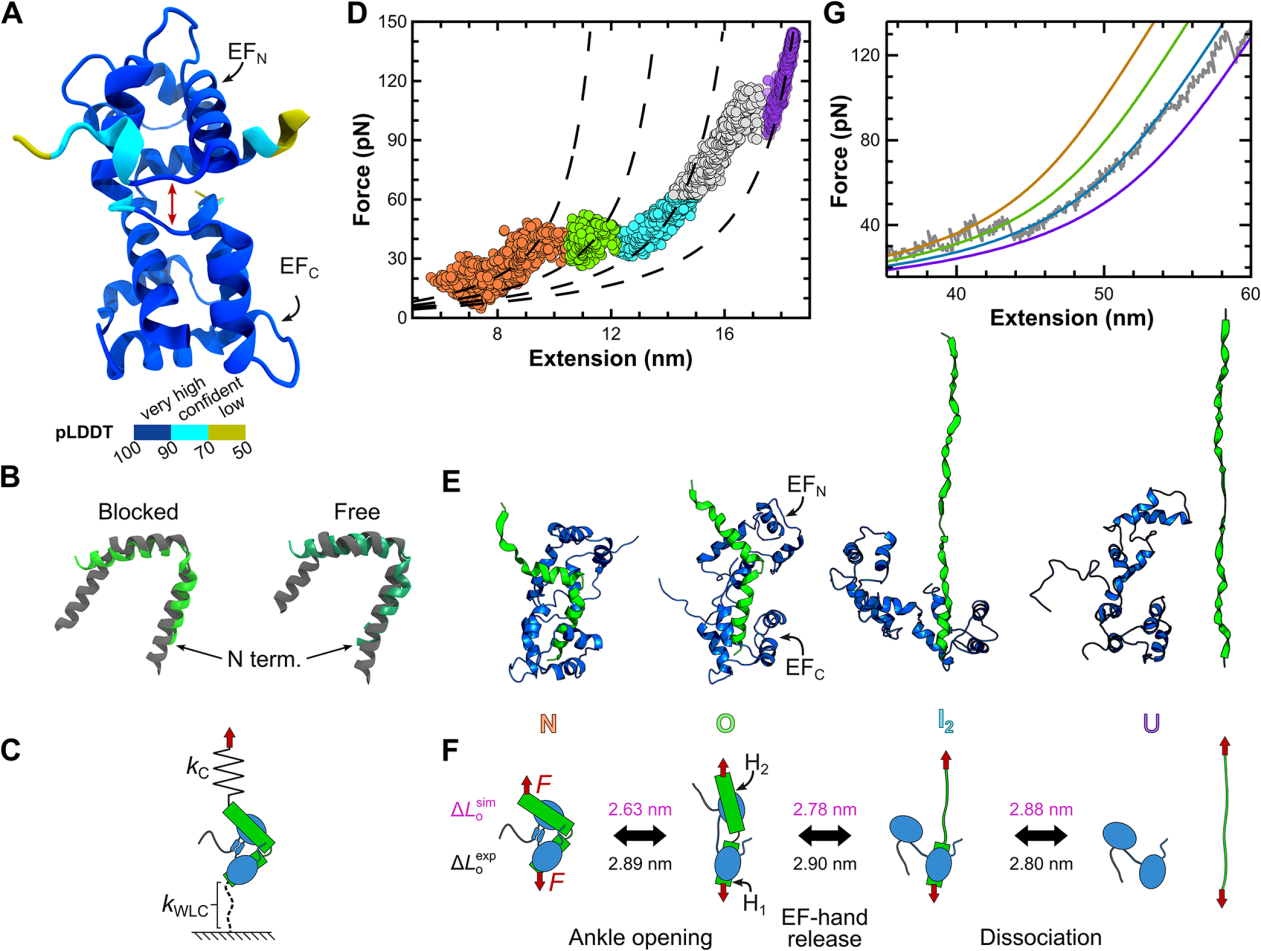
Structural insights derived from coarse-grained simulations. (A) A ribbon diagram of the RLC complex as predicted by AlphaFold 3, where the color code represents the degree of confidence in the residue location (pLDDT).^56^ The CALLI interaction (red arrow) between the N-terminal tail immediately before RLC’s canonical first helix (helix A) and the peptide linker between the two EF-hand domains is predicted with confidence to very high confidence, with 70% of the CALLI-forming residues predicted with very high confidence. Note, regions predicted with very low confidence (pLDDT < 50) are not shown. (B) Ribbon diagram of the simulation starting state for the lever-arm α-helix (green) superimposed with the lever arm for the OFF state (gray, PDB: 8ACT^17^) in the vicinity of the LA^RLC^ for the blocked and free head (left and right, respectively). (C) A conceptual diagram of the coarse-grained simulations showing the N-terminus of the LA^RLC^ restrained via a virtual WLC (*k*_WLC_) spring and pulled from the C-terminal by a Hookean spring representing the AFM cantilever (*k*_C_). (D) Ten simulated force-extension curves at *v*_sim_ = 40 μm/s showing two intermediates with superimposed fits to the WLC model to derive the change in contour length (Δ*L*_0_) between states. Note, the second intermediate was not well described by the WLC model. (E, F) Snapshots of molecular configuration and structural cartoons that detail the four states in the unfolding pathway containing the native (N), open ankle (O), intermediate (I_2_) and unfolded (U) states. The three transitions correspond to ankle opening (N→O), EF-hand release (O→I_2_) and RLC dissociation (I_2_→U). The Δ*L*_0_ for each transition derived from simulation and experiment are shown. (G) An experimental force-extension curve with superimposed WLC curves using the Δ*L*_0_ derived from the simulations, where U is the reference state to account for variations in PEG-linker length. The overlap of the simulation-derived curves with the experimental data provides confidence in using molecular configurations derived from the simulations.

The AlphaFold 3 predicted ankle angle matched the ankle angle for the blocked and free head in the IHM state for human β-cardiac myosin^17^ (Figure 4B green vs gray, respectively). Figure 4B also highlights the mechanical strain exerted on the RLC complex by the added kink in the lever-arm just C-terminal to the LA^RLC^ in the IHM state (Figure 4B). We note that the loop–loop interaction in CALLI is resolved in the IHM of human β-cardiac cryo-EM structure, but somewhat distorted in the free head with the remaining portion of the N-terminal tail being unresolved (residues 1–19, Figure S5).^17^

To better compare molecular configurations of simulated intermediates to observed intermediates in the force-extension curves, the N-terminal amino acid of the LA^RLC^ was held stationary via a spring modeled as a worm-like chain^44,45^ (WLC) [contour length (*L*_0_ = 6 nm) and persistence length (*p* = 0.4 nm)]. The other end of the molecule was retracted at a fixed velocity via a Hookean spring with a stiffness (*k* = 8 pN/nm) on par with our FIB-modified cantilevers (Figure 4C). Importantly, 10 of the 27 simulations showed two intermediates, with force-extension curves strikingly similar to the experimental data (Figure 4D). This similarity provided initial confidence that these coarse-grained simulations were capturing the essential inter- and intramolecular interactions of the RLC complex unfolding under force.

The resulting molecular configurations from these simulations provided insight into the two intermediates. In particular, the first intermediate corresponds to a conformational change in the ankle rather than a force-induced unfolding. The ankle angle goes from an acute angle in the native state (N) to an extended conformation, denoted as the open ankle state (O) (Figure 4E,F). The next transition corresponded to the unfolding of the C-terminal α helix (H_2_ as defined in Figure 2a) of the LA^RLC^ with the associated displacement of the RLC’s N-terminal EF-hand domain (EF_N_). We denote this state as I_2_. This second intermediate was an obligate intermediate in all 27 unfolding trajectories. At significantly higher forces, the C-terminal EF-hand domain (EF_C_) was displaced from H_1_, resulting in the fully unfolded state (U).

We compared the simulations with experimental data by leveraging the WLC characteristics of the simulated force-extension curves via a change in contour length (Δ*L*_0_) analysis. Importantly, this comparison did not require matching rupture forces since differences in rupture forces between simulation and experiment arise from the 67-fold difference in pulling speed and/or the details of the model’s force-field parametrization.^55,57^ On the other hand, comparison based on Δ*L*_0_ focused on a change in the molecular configuration. To determine Δ*L*_0_, we first segmented the 10 curves showing two intermediates into the four observed states (N, O, I_2_ and U). As discussed above in the definition of a state, N, O, and U segmentation was based on each segment being well described by the WLC model that upon fitting yielded *L*_0_ for each state. In contrast to these three states, state I_2_ was not well described by WLC model (Figure 4D, dashed lines). The origin of this discrepancy is discussed in the next section. To determine *L*_0_ for state I_2_, we only fit the initial portion of its trajectory that reflected the entry into I_2_. With these four values of *L*_0_, we determined the three values of Δ*L*_0_.

Superimposing these sets of simulated changes in Δ*L*_0_ onto an experimental force-extension curve (*v* = 600 nm/s) allows for comparison while accommodating differences in *L*_0_ due to variations in PEG length and tip attachment location (Figure S6). As shown in Figure 4G, the agreement was excellent. Hence, this set of computationally predicted Δ*L*_0_ captured the conformation states seen in the experiment, allowing us to assign molecular configurations to these states with confidence. We next directly compared the computationally predicted and experimentally deduced Δ*L*_0_ for the three transitions: N→O [2.63 ± 0.06 nm (fit ± SD) vs 2.89 ± 0.06 nm (mean ± SEM, *N* = 72), respectively]; O→I_2_ [2.78 ± 0.03 vs 2.90 ± 0.06 (*N* = 72)]; and I_2_→U [2.88 ± 0.02 vs 2.80 ± 0.04 (*N* = 96)] (Figure 4F), where the standard deviation in each fit is the square root of the diagonal of the covariance matrix. This comparison also shows excellent agreement. The differences between the computational prediction and the experimental result were less than the change in contour length associated with a single amino acid (0.365 nm/aa).^58^

### Simulation Highlights Time Evolution of Key Contacts during Unfolding

The close correspondence between experiment and coarse-grained simulations enabled us to investigate the changes in intra- and intermolecular contacts as the RLC complex proceeded through its unfolding pathway. To do so, we analyzed a simulated unfolding trajectory that shows back-and-forth transition between N and O (Figure 5). As expected, the force dropped during N→O transitions and increased during O→N transitions (Figure 5A, see also Movies S2 and S3). After two cycles of these back-and-forth transitions, the molecule goes into I_2_, with a slightly larger force drop. As the force continues to increase, the C-terminal EF-hand domain released during the I_2_→U transition.

**Figure 5:**
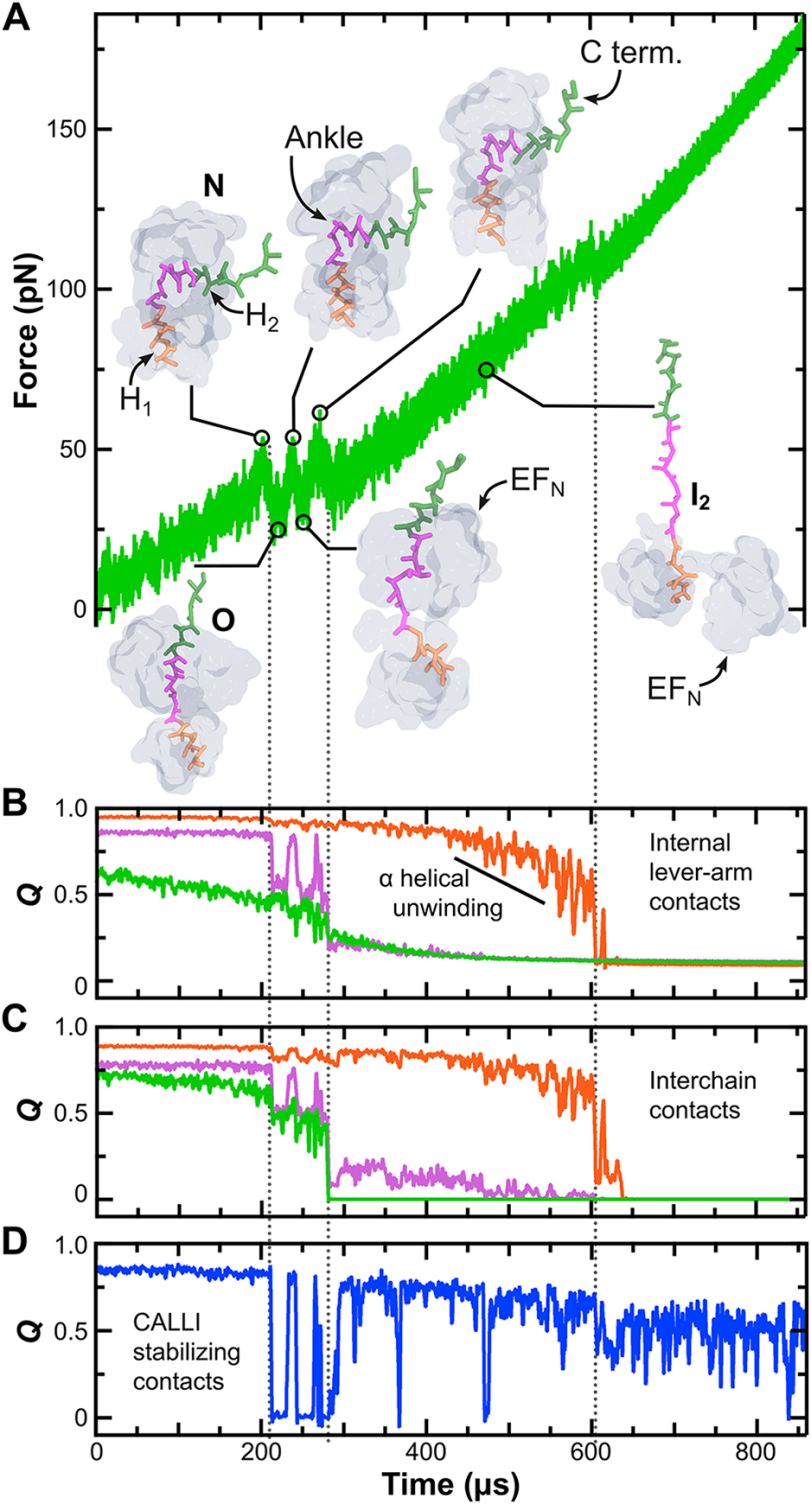
Changes in structural contracts during ankle opening and initial EF-hand release: (A) A force-vs-time trace from the coarse-grained simulation shows back-and-forth transitions between the native (N) and the open-ankle state (O) followed by the release of EF_N_ (I_2_) and eventually the full dissociation of the RLC (U). Inset are snapshots of the molecular configurations from the simulation, where the RLC is shown as a transparent molecular surface. The LA^RLC^ is displayed in three colors corresponding to the ankle (magenta) and H_1_ and H_2_ (orange and green, respectively) that interact with the RLC’s EF-hand domains (EF_C_ and EF_N_, respectively). (B–D) Fraction of native contacts (*Q*) as a function of time determined for the contacts within the LA^RLC^ for each color-coded region [H_1_, ankle, and H_2_ (panel B)], contacts between each segment of the LA^RLC^ and the RLC (panel C), and selected CALLI stabilizing contacts (panel D). The ankle-associated magenta curves show three distinct values that correspond to the three states: N, O, and I_2_. The H_2_-associated green curves show smaller variations upon transition between N and O along with a general decrease with increasing force followed by an abrupt drop upon entering I_2_ as seen in panel C. Note, *Q* does not start at 1 due, in part, to starting the simulation at a preloaded force of ∼5 pN. The H_1_-associated orange curves show most internal LA^RLC^ contacts were preserved during the N→O transition but dropped at higher force in I_2_ due to unraveling of H_1_, providing a molecular explanation for why I_2_ is not well described by the WLC model. The three dotted lines represent transitions from N→O, O→I_2_, and I_2_→U (left to right, respectively).

We note that these back-and-forth transitions illustrate the benefit of coarse-grained simulations beyond the simple reduction in computational cost. Coarse-grained simulations allow for much longer simulation times where such near-equilibrium conformational dynamics can occur. Moreover, they also allow for domain-scale reorganization—represented here by ankle opening—that happens on time scale longer than accessible in a typical steered molecular dynamics simulation.^59^ Stated differently, in a simulation using a typical all-atom pulling velocity (0.25 m/s),^27^ EF_C_, EF_N_, and the ankle angle might be expected to be effectively fixed on a ∼1-μs time scale, resulting in H_1_ and/or H_2_ unwinding before ankle opening could occur. As a result, simulations with such high pulling speeds would likely predict a dramatically different unfolding pathway.

For analyzing inter- and intramolecular contacts, we divided the LA^RLC^ into three domains, H_1_ (Ser^810^–Ala^820^), ankle (Phe^821^–Phe^834^) and H_2_ (Lys^835^–Glu^846^), shown as orange, magenta, and green, respectively (Figure 5A). Native contacts were defined as those starting within 8 Å. Contacts were broken once they extended by 2 Å.^55^ To look at the change in native contacts, we used *Q*, the fraction of native contacts with nearest neighbor contacts excluded. Sudden changes in *Q* can be interpreted as discrete transitions between intermediates.

We first analyzed the intramolecular contacts within each domain of the LA^RLC^ (Figure 5B). The internal ankle contacts (magenta) underwent discrete changes as the complex went back and forth between N and O, with a further loss of intra-ankle contacts as the complex went from O to I_2_. The intramolecular contacts within H_2_ (green) did not start at *Q* = 1 because the last 6 aa of the LA^RLC^ were not well structured at zero applied force and the simulation was preloaded at ∼5 pN.

Applying this analysis to the intramolecular contacts of H_1_ provides an explanation for the non-WLC behavior seen for I_2_ in Figure 4D. Initially, *Q* stays at approximately 1, consistent with a well-formed α-helical structure (Figure 5B, orange). But, upon entering I_2_, *Q* continues to stay at ∼1 before monotonically decreasing to a mean value of ∼0.5, followed by an abrupt drop upon entering U. This monotonic decrease in *Q* arises from a gradual unraveling of the H_1_ α helix. Such α-helical unwinding is not modeled by a single *L*_0_ and is therefore the reason I_2_ is not well described by the WLC model. As a result, we used the onset of this monotonic decrease in *Q* as the high force limit for fitting the WLC to I_2_.

Discrete changes in *Q* occurred when analyzing interactions between the LA^RLC^ and the RLC (Figure 5C). The ankle-mediated interactions again underwent large discrete changes between N, O, and I_2_. The interchain contacts between EF_N_ and H_2_ were slightly more stable in N than the intrachain lever-arm ones and showed stepwise decrease upon entering O and I_2_. The interchain contacts in H_1_ were also slightly more stable in I_2_ and showed a large single step upon entering U.

Visual inspection of the molecular conformations suggested that ankle opening would affect CALLI stabilizing contacts. We therefore cataloged intra-RLC contacts stabilizing CALLI (Met^20^–Gln^25^ & Leu^90^–Pro^95^) (Table S4). *Q* for these contacts indeed underwent large changes upon the opening and closing of the ankle (N↔O) (Figure 5D). Note, the high, yet fluctuating value for *Q* in Figure 5D after entering I_2_ resulted from the detached EF_N_ domain undergoing tethered Brownian motion immediately adjacent to the bound EF_C_ domain and the reformation of the CALLI interface while EF_N_ was not bound to H_2_ (Movie S4). This transient reformation should not be overinterpreted because it arises, in part, from the bias to reform the native contacts, including CALLI contacts, in a structure-based, coarse-grained simulation (see below for a more complete description of the limitation of such models).

### Force Propagates through CALLI Despite Pulling Across Lever Arm

Where does the mechanical rigidity of the ankle arise? We hypothesized that CALLI was stabilizing the native state (Figure 2A, bottom left). We illustrate the complementary nature of the molecular surfaces of the two loops making up CALLI by showing an all-atom molecular surface of a slightly larger region of the N-terminal domain [Met^20^–Gln^25^ (Figure 2A, bottom right)].

To investigate this hypothesis, we deduced the optimal force-propagation pathway by adapting an analysis previously developed for all-atom steered molecular dynamics^27^ to our coarse-grained simulations. In this analysis, the force between atoms or, in our case, interaction centers leads to a tauter, stiffer linkage that, in turn, leads to increased correlated motion (see Supporting Information for details). To implement this analysis, we analyzed an unfolding trajectory in the native state but close in force to the transition into the open state (*F*_sim_ ≈ 27–32 pN). The optimal force-propagation pathway is denoted as a red tube in Figure 6A. Starting from the N-terminal of the LA^RLC^, the force propagates about one helical turn along the lever arm (Ser^810^–Val^813^) until transitioning to the RLC—including directly through our hypothesized loop–loop interaction forming CALLI (Asp^94^↔Phe^21^)—and then through helix A of the RLC (Gln^25^), also part of CALLI. The pathway returns to the lever-arm at Lys^837^. This analysis highlights the importance of CALLI in stabilizing the native state at an acute ankle angle.

**Figure 6:**
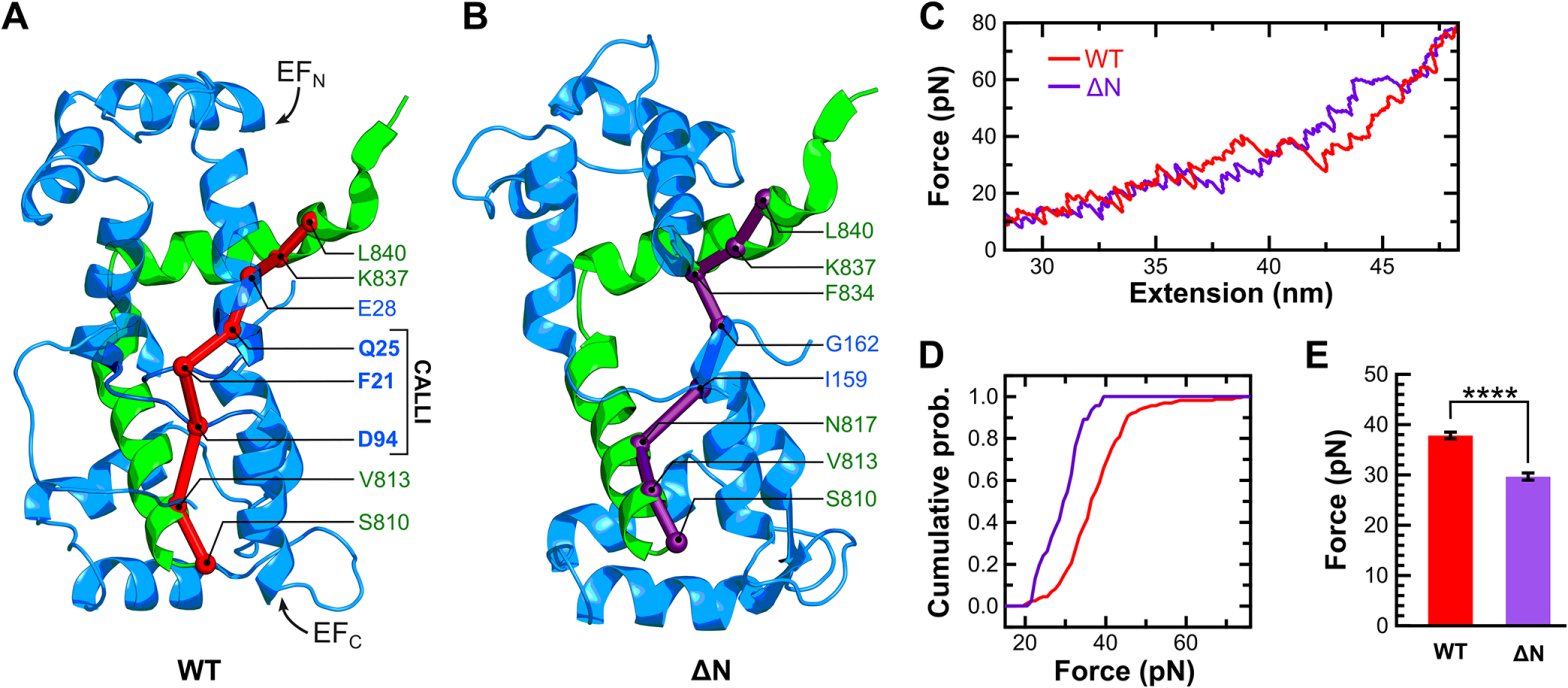
CALLI stabilizes the acute ankle angle. (A) A ribbon diagram shows the optimal force-propagation pathway when pulling across the LA^RLC^. This pathway is denoted with a red tube. Amino acids on the force-propagation pathway are shown (green = LA^RLC^, blue = RLC) with those within CALLI shown in bold text. Starting from the bottom, the force initially propagates within the lever arm helix (H_1_) and then passes directly through CALLI before reconnecting with the lever arm near the end of H_2_. (B) A ribbon diagram shows the altered optimal force-propagation pathway (purple) for the Δ*N* construct, which deletes the N-terminal tail prior to the start Helix A of the RLC (technically residues 2–22). Note, this diagram is slightly rotated from panel A to allow for better visualization of residues along this altered pathway. (C) Two force-extension curves comparing a wild-type (WT) and a Δ*N* construct (red vs purple, respectively). The cantilever was retracted at 5000 nm/s to facilitate detection by increasing the ankle-opening force. (D) Cumulative probability distribution for ankle-opening force comparing the WT and Δ*N* constructs shows the RLC’s N-terminal tail contributed to stabilizing the native state against ankle opening (*N* = 161 and 46, respectively). Note, this data was acquired with the same individual cantilever to improve precision by avoiding uncertainties associated with comparing distributions acquired with different cantilevers.^60,61^ (E) The mean opening ankle-opening force plotted for the WT and Δ*N* constructs [37.8 ± 0.7 pN (mean ± SEM; *N* = 161) vs 29.6 ± 0.7 pN (*N* = 46), *p* < 10^–4^].

To computationally test this predicted importance of CALLI, we removed one of the two loops participating in CALLI by deleting the RLC’s N-terminal tail (Δ*N*). Technically, we deleted residues 2–22 due to the requirement for an expressed protein to start with a methionine. AlphaFold 3 was used again to predict the structure, which showed an essentially identical overall fold for the RLC complex, except for a slight shift in the start of helix A of the RLC (Figure S7). We next simulated 18 trajectories of the Δ*N* variant using the same parameters used for the wild type (WT) simulation. The Δ*N* trajectories showed rapid back-and-forth ankle opening (N↔O transitions) in all 18 simulations. In contrast, only 10 out 27 the WT unfolding trajectories contained a N→O transition, presumably because the other trajectories exited from the native state at a sufficiently high force such that the O state was not transiently occupied. As expected, the computationally predicted initial ankle-opening force was reduced for the *Δ*N construct compared to WT construct [12.9 ± 1.3 pN (mean ± SEM, *N* = 18) vs 41.5 ± 0.9 pN (*N* = 10)] (Figure S8A–C). These absolute values are less important than that the Δ*N* simulations predicted both that the Δ*N* construct still has a mechanical transition from N→O and that this transition has a lower ankle opening force, within the limitations of these coarse-grained simulations, as discussed more fully below.

We next showed that optimal force-propagation pathway for the Δ*N* construct was significantly different from the WT construct (Figure 6B). In particular, it no longer passes through CALLI. Rather, this analysis shows that the force propagates approximately an extra helical turn along both H_1_ and H_2_ (adding residues Asn^817^ and Phe^834^, respectively) while only briefly going through helix H of the RLC (Ile^159^ and Gly^162^).

### N-terminal Tail of the RLC Stabilizes the Native Acute Ankle Angle

To experimentally test the importance of CALLI, we expressed the Δ*N* construct simulated above. Neglecting the obligate initial N-terminal methionine, this new RLC construct started at the beginning of helix A (residue 23),^12^ presumably abolishing the CALLI contribution to ankle stabilization. To better detect small transitions at low forces, we pulled at an even higher velocity (5,000 nm/s) to increase the rupture force. Individual force-extension curves still often showed two intermediates for Δ*N* construct but at a lower ankle-opening force than similarly acquired curves for the WT RLC (Figure 6C, purple versus red, respectively). To more precisely compare ankle-opening force between these two protein constructs, we acquired a set of data for the WT and Δ*N* constructs taken with a single cantilever, a technique pioneered by Hermann Gaub’s lab^60^ that avoids uncertainties associated with comparing distributions acquired with different cantilevers.^61^ The resulting cumulative probability distributions of the ankle-opening force show a clear reduction in ankle opening for the Δ*N* construct compared to WT (Figure 6D). We established statistical significance (*p* < 10^–4^) by comparing the mean ankle-opening force for Δ*N* and WT constructs [29.6 ± 0.7 pN (mean ± SEM; *N* = 46) vs 37.8 ± 0.7 pN (*N* = 161)] using a two-tailed Mann–Whitney test (Figure 6E). Hence, experimentally, CALLI makes a significant contribution to stabilizing the human β-cardiac myosin RLC complex in its acute ankle angle in conjunction with other stabilizing contacts within the RLC complex (Figure 5B,C).

### The Angular Location of the Transition State for Ankle Opening

Single-molecule force spectroscopy is able to probe parameters of the free-energy landscape stabilizing particular molecular configurations.^62,63^ In an initial characterization, we sought to determine the distance to the transition state (Δ*x* ^‡^) by measuring the mean rupture force as a function of loading rate (∂*F*/∂*t*) using dynamic-force spectroscopy.^25,26^ From this experimentally deduced Δ*x* ^‡^, the angular rotation to the transition state (Δθ^‡^) can be estimated with the aid of the coarse-grained simulations.

To acquire a large number of ankle-opening events, we repeatedly probed individual molecules tens of times. To do so, we first fully retracted the cantilever, breaking the cohesin-dockerin interaction with the unfolding of the GB1 marker domains used to verify single-molecule attachment. The tip was then brought back into gentle contact with the surface to initiate reattachment and then retracted to a high force (*F* = 300 pN) to unfold both GB1 domains and reverify a single-molecule attachment. The tip was again brought down to just above the surface (*z* = 0–5 nm) and briefly (3 s) held there to promote GB1 refolding. The cantilever was cyclically retracted at seven target velocities from 100 to 10,000 nm/s up to a second, lower force (80 pN) sufficient to open the ankle and returned to the surface for a shorter zero-force dwell (1 s) sufficient for complex to return to the native state (Figures S9 and S10). Each repeated set of seven velocities occurred over ∼10 s, within the period over which our cantilevers showed sub-pN stability (Figure S2A).^38^ After each set, the cantilever was fully retracted to break the cohesin-dockerin linkage and the process was started again. Comparing the initial ankle-opening force with a repeated ankle opening of the same molecule was justified because the initial ankle-opening force from 40 individual molecules was indistinguishable from repeated unfolding of the two molecules a total of 40 times [34 ± 1 pN (mean ± SEM) vs 35 ± 1 pN @ *v* = 1000 nm/s] (Figure S11).

As the Bell-Evans model is formulated to analyze a single, irreversible barrier crossing per force probe retraction not near-equilibrium transitions, we only analyzed the first N→O transition per cantilever retraction. Plotting the resulting rupture force as a logarithmic function of loading rate yielded approximately linear behavior when analyzing traces showing N→O transitions (Figure 7A). A Bell-Evans analysis yielded Δ*x* ^‡^ = 0.58 ± 0.09 nm (fit ± SD).

**Figure 7:**
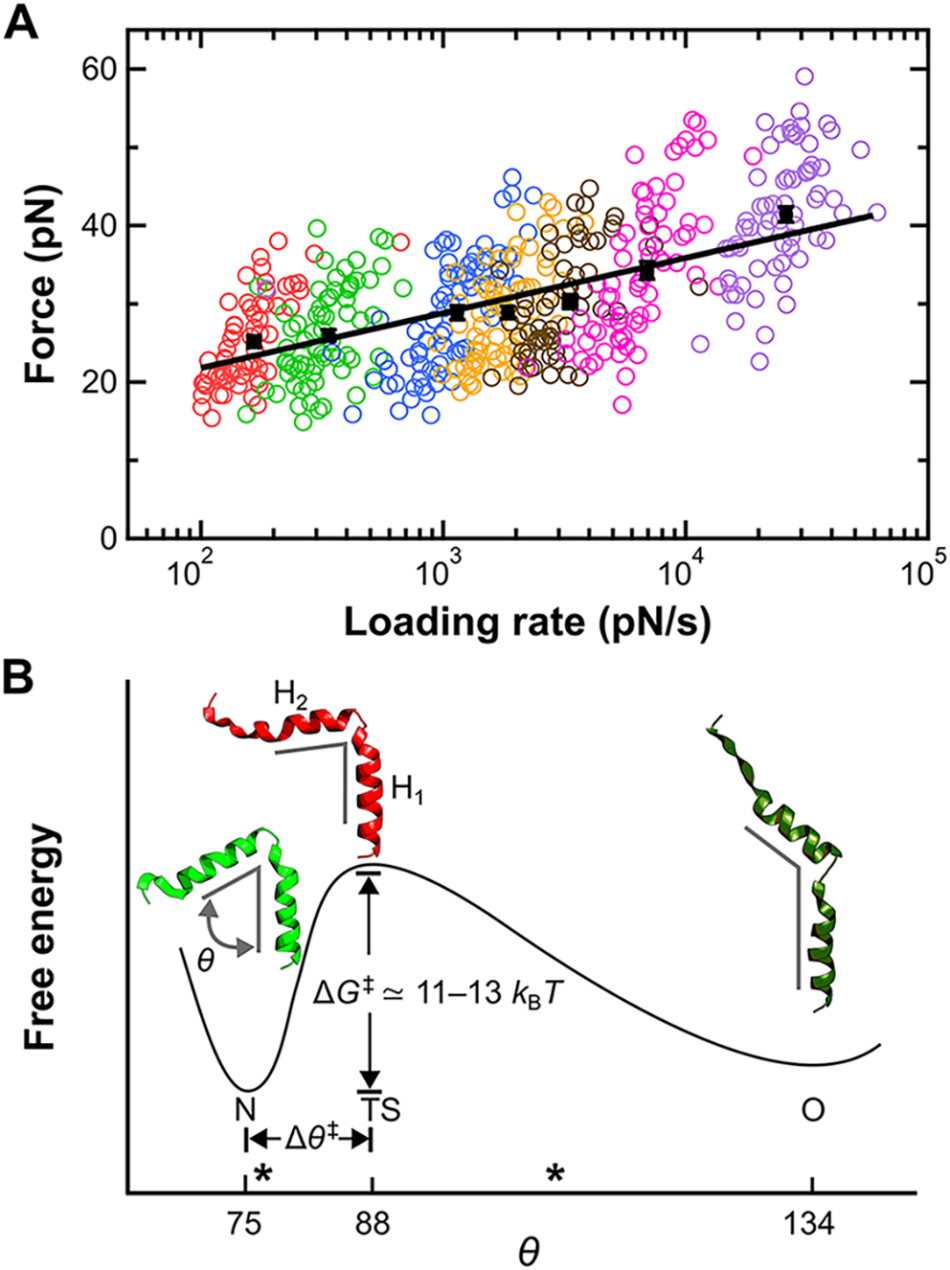
Dynamic force spectroscopy analysis of ankle opening. (A) Average ankle-opening force plotted as a function of loading rate for identified N→O transitions (black) where error bars represent the SEM. Individual molecular openings are color coded by pulling velocity [red (100 nm/s, *N* = 63), green (200 nm/s, *N* = 68), blue (600 nm/s, *N* = 73), orange (1000 nm/s, *N* = 66) brown (1600 nm/s, *N* = 60), magenta (3000 nm/s, *N* = 69), and violet (10,000 nm/s, *N* = 69)]. Analysis of this data with a Bell–Evans model (line) yielded the distance to the transition state [Δ*x* ^‡^ = 0.58 ± 0.09 nm (fit ± SD)]. (B) An illustrative free-energy landscape for ankle opening as a function of ankle angle (θ), where N is the native state (θ = 75°), TS is the transition state (θ = 88°), and O is the open ankle state (θ = 134°). The height of the free-energy barrier (Δ*G* ^‡^) is estimated to be 11–13 *k*_B_ *T*. The asterisk symbols (*) represent the two ankle angles observed for scallop smooth muscle myosin. Ribbon diagrams of these states captured from coarse-grained simulation. For clarity, the RLC is not shown.

A better coordinate for discussing ankle opening is the ankle angle (θ), which starts at θ = 75° in the native state under zero load. To do so, we defined θ from our coarse-grained simulations based on the angle between the vectors defined by the two lever arm helices H_1_ and H_2_. Our estimate for the molecular configuration of the transition state was deduced from the simulated unfolding trajectory by using the configuration that had an averaged increase in molecular extension corresponding to Δ*x* ^‡^. This simulated extension was often just at the cusp of the rapid increase in extension as the complex transitioned from N to O, suggesting that it indeed approximated the location of the transition state (Figure S12A). We then determined the averaged location of the transition state by applying this analysis to the ten simulations showing N→O transitions, yielding θ = 88 ± 2° (mean ± SD) (Figure 7B) and therefore the angular rotation to the transition state [Δθ^‡^ = 13 ± 2° (mean ± SD)]. This relatively small angular change to the transition states results from the rupture of the CALLI contacts stabilizing the native state (Figures 5D and S12C). This ankle-opening analysis is shown at higher temporal resolution in Figure S12D–F.

More broadly, this result suggests a force-induced mechanical transition in the ankle for the human β-cardiac myosin RLC complex that is distinct from that in scallop smooth muscle myosin, since the ankle opening for the human β-cardiac myosin ankle requires force and the transition state occurs between these two states resolved for scallop [θ = 77 and 109° (denoted as * in Figures 7B)].^12^ [Note, the same two ankle angles and overall RLC structures were observed for scallop smooth muscle myosin even when the RLC was phosphorylated,^64^ where such phosphorylation acts as switch by destabilizing the IHM or OFF state rather than changing the isolated RLC complex structure.^17^] The average ankle angle in the open state after opening under force was 134° (*N* = 3) as deduced from trajectories showing back-and-forth N↔O transitions (Figure S12B). We note that the open-ankle angle was not tightly confined but underwent relatively large angular fluctuations [140 ± 11° (mean ± SD) @ 28–30 pN] and shifted to a larger mean value with increasing force (149 ± 11° @ 43–46 pN) (Figure S13). In contrast, ankle angle was relatively tightly confined in the native state and native ankle angle shifted only slightly from zero force to 17–20 pN (77 ± 3° @ 17–20 pN), where this analysis was performed on 17.6-μs trajectory segments smoothed over 100-ns window using a second-order Savitzky–Golay filter.

Within the framework of our current coarse-grained simulations, we asked whether this θ = 88 ± 2° was a reasonable estimate of the transition state location by computing the fraction of persistent native contacts (*Q*_ankle_) that break upon ankle opening (see Methods for details). We used *Q*_ankle_ = 0.5 as an operational proxy for the transition state. Using this definition, we find that *Q*_ankle_ = 0.49 at location of transition state (θ = 88°) for a single trajectory (Figure S14). Reversing this process, we determined the angular location where *Q*_ankle_ = 0.5 for the 10 trajectories containing an N→O transition and deduced θ = 86 ± 2° (mean ± SD), in close agreement with the first estimate θ = 88 ± 2° (mean ± SD), which used Δ*x* ^‡^ deduced from the dynamic-force-spectroscopy assay. Future simulations using the kinetic committor *P*_fold_ can refine this estimate.^57,65^

Finally, we estimated the height of the 1D free-energy barrier (Δ*G* ^‡^) for the N→O transition using the near-equilibrium fluctuations shown in Figure 3C. The time scale for the back-and-forth transitions at *F* ∼ 30 pN was 1 ms (*k* = 1000 s^–1^). To estimate Δ*G* ^‡^, we used the Arrhenius equation *k*(*F*) = *A*exp­[−(Δ*G* ^‡^ – *F*Δ*x* ^‡^)/*k*_B_ *T*] where *A* is the attempt frequency, *k*_B_ *T* is the thermal energy, and Δ*x* ^‡^ = 0.58 nm, as derived from the Bell-Evans analysis. This analysis yielded Δ*G* ^‡^ ≈ 11–13 *k*_B_ *T* when estimating *A* to be on the order of 10^6^–10^7^ s^–1^,^66^ typical of values assumed in such analysis.^67^ We note that this simple 1D free-energy landscape assumes that all the other degrees of freedom are fluctuating fast relative to the slow diffusion along θ across the 1D free-energy barrier. Future work can investigate if ankle opening/closing may be better described by a multidimensional free-energy landscape, where an obvious added dimension would be a second angle (φ), where φ represents the twist angle between H_1_ and H_2_ helices.

### Different Pulling Geometries Yield Different Unfolding Pathways

We developed two other pulling geometries (Figure 2B,C) to complement pulling across the LA^RLC^. These alternative pulling geometries isolated different interactions within the RLC complex and have been explored in previous single-molecule force-spectroscopy studies of the structurally related calmodulin.^47,68,69^ In the first alternative construct, we isolated the force application to across the RLC by applying force to its N and C termini. The resulting force-extension curves showed a single intermediate at *v* = 1000 nm/s (Figure 8A,B). Conceptually and computationally, there are two alternative unfolding pathways where either the EF_C_ or the EF_N_ domain unfolds first [Pathway 1 and 2, respectively (Figure 8A,C)]. Experimentally, the resulting intermediate in the force-extension curve was well described by a WLC model, consistent with the unfolding of one of the two EF-hand domains.

**Figure 8:**
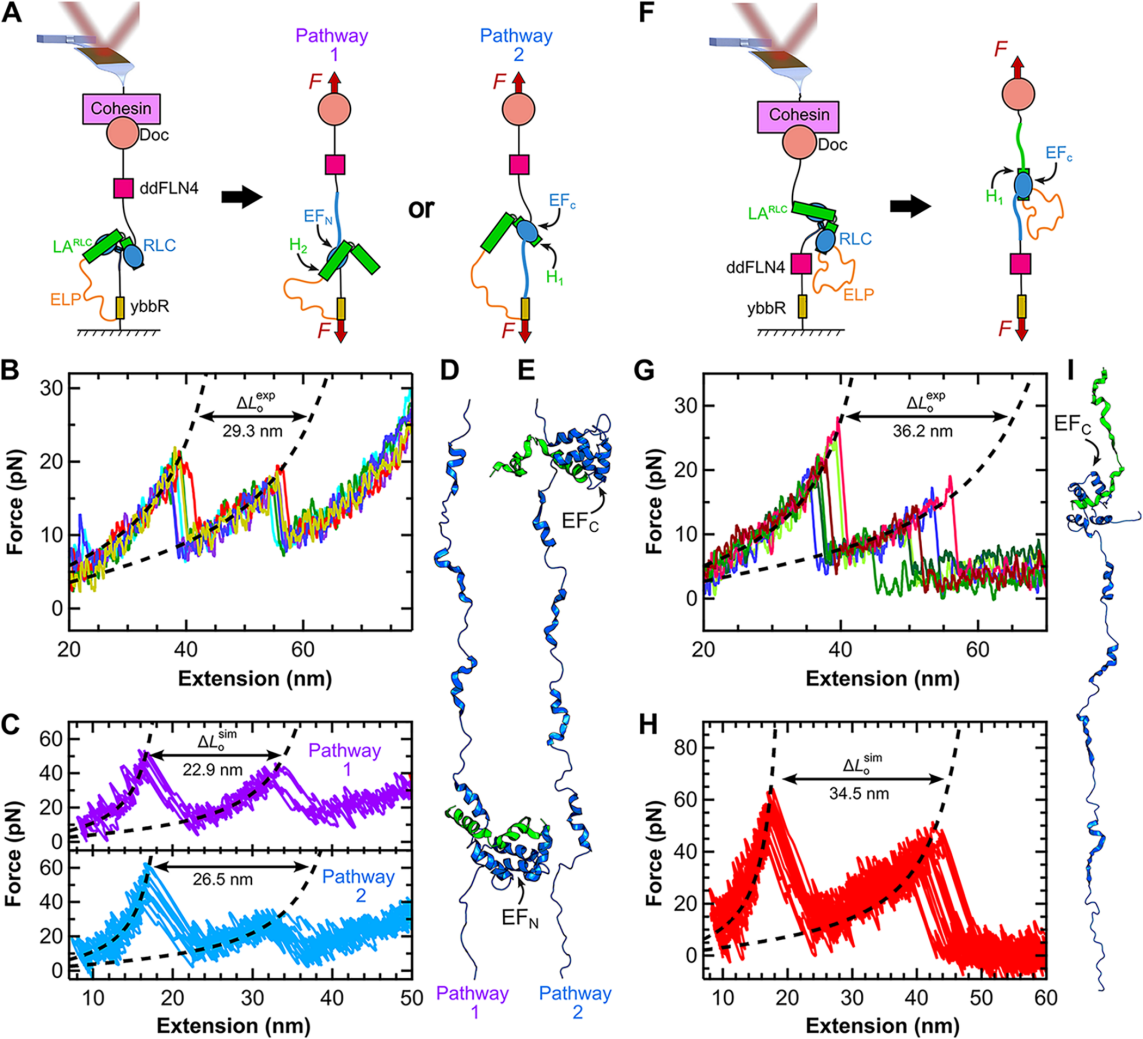
Different unfolding pathways for different pulling geometries. (A) Illustrations of the polyprotein show the native and the two computationally predicted intermediate states when pulling across the RLC by applying force to its N- and C-termini. (B) Six representative force-extension curves when pulling at 1000 nm/s show a single intermediate with superimposed WLC force-extension curves, where a fit to the native state determined *L*_0_ and then the average experimentally determined Δ*L*_0_ was used to generate the WLC model for the intermediate. (C) Simulated force-extension curves sorted by the unfolding pathway with the computationally predicted Δ*L*_0_ for each pathway deduced by WLC fits to the data. (D, E) Molecular snapshots from the simulations show that the intermediate can arise from unfolding either the EF_C_ domain (5 out of 18) or the EF_N_ domain (12 out of 18), with one trajectory showing a more complicated unfolding pathway. (F) Structural illustrations of the polyprotein show the native and intermediate states when pulling across the RLC complex by applying force to the RLC’s N-terminus and the C-terminus of the LA^RLC^. (G) Six force-extension curves in this pulling geometry show a single obligate intermediate in the unfolding pathway. (H) Force-extension curves from the 18 coarse-grained simulations of this pulling geometry also revealed a single intermediate. The WLC fit to the intermediate was done at the higher force region due to partial H_1_ unwinding at lower forces that may be masked in the experimental records due to the low forces (<10 pN). (I) A molecular snapshot from the simulation shows the intermediate arose from the rupture of the H_2_:EF_N_ interaction concurrent with the force-induced unfolding of each of these elements. Experimental data smoothed to 1 kHz and aligned laterally to the native state to accommodate variations in PEG length.

Interestingly, coarse-grained simulations of this pulling geometry yielded force-extension curves that also showed one intermediate arising from an EF-hand domain unfolding (Figure 8C), but which EF-hand domain unfolded was not necessarily fixed (Movie S5). Among 18 unfolding trajectories, EF_C_ unfolded first in five simulations (Figure 8C, pathway 1) while EF_N_ unfolded first in the other 12 trajectories (Figure 8C, pathway 2) with one trajectory showing a more complex unfolding pathway. Molecular snapshots of these two intermediates are shown in Figure 8D,E. The fact that EF_C_ could unfold first at all was surprising given how much more force had to be applied across the lever arm to mechanically dissociate the H_1_:EF_C_ interaction compared to the H_2_:EF_N_ interaction both experimentally and in simulations when pulling across the LA^RLC^ (Figures 3A, 4D, and S15). Thus, the H_1_:EF_C_ interaction is particularly robust to mechanical forces applied across the lever arm, showing again that mechanical resilience depends on the direction of applied force.^70,71^

A contour-length analysis is suggestive that the experimentally observed unfolding pathway is Pathway 2, where the H_2_:EF_N_ interaction unfolds first. However, we first note the unfolding forces from the native and intermediate state are quite low (15–20 pN @ 1000 nm/s), a low force by AFM standards.^37^ As a result, there is less curvature than in force-extension curves going up to higher forces, making it more difficult to accurately constrain *L*_0_ and thereby Δ*L*_0_. Our experimentally derived Δ*L*_0_ is 29.3 ± 0.4 nm (mean ± SEM; *N* = 66). Applying this analysis to the two computationally observed unfolding pathways yielded Δ*L*_0_ = 22.9 ± 0.1 nm (mean ± SD; *N* = 5) for pathway 1 and Δ*L*_0_ = 26.5 ± 0.1 (*N* = 12) for pathway 2 (Figure 8C). The experimental value is closer to computationally predicted value for pathway 2, but our concerns with drawing firm conclusions based on fitting these low-unfolding force transitions persist. We note that we do not have the same degree of absolute agreement shown in Figure 4F, since the Δ*L*_0_ for these EF-hand-domain unfolding transitions are ∼10-fold larger.

In the second alternative pulling geometry, we pulled across the RLC complex by applying force to the N-terminus of the RLC and C-terminus of the LA^RLC^ (Figure 8F). The resulting force-extension curves showed one obligate intermediate (Figure 8G). This intermediate was well modeled by a fit to the WLC model. Coarse-grained simulations showed this intermediate arose from the rupture of the H_2_:EF_N_ interaction coupled with the concurrent force-induced unfolding of both the H_2_ helix and the EF_N_ domain (Figure 8H,I; Movie S6). We fit the simulated 18 force-extension curves to the high-force region of the intermediate due to a small deviation from WLC model in the simulated traces at lower forces that were unresolved in the experimental traces. The resulting Δ*L*_0_ derived from the simulation again matched the experimental data [Δ*L*_0_ = 34.5 ± 0.1 (fit ± SD; *N* = 18) and Δ*L*_0_ = 36.2 ± 0.3 (mean ± SEM; *N* = 68), respectively]. Looking forward, these different pulling geometries may yield insights into HCM-causing mutations that change the local interaction strength between the RLC and the LA^RLC^, a mechanism of action distinct from a change in ankle stiffness or orientation.

### Limitations to Coarse-Grained Models

Notwithstanding the structural insights that we derived from the coarse-grained simulations, such models have their limitations. Beyond the obvious lack of atomic-scale descriptions and explicit solvent interactions, coarse-grained models generally have structure-based energy terms derived from atomistic reference structures (e.g., OLIVES^72^ and GoMartini^49^ for MARTINI3 force field) that stabilize the native fold. The human β-cardiac RLC has unstructured N- and C-terminal tails [residues 1–16 and 162–166 using AlphaFold 3′s prediction of not being in a secondary structural element (Figure S4)], implying these regions are flexible. So, conformational heterogeneity or transient interactions of these tails that stabilize the acute ankle angle in the N state may therefore be underestimated. In coarse-grained simulations, these interactions have to be modeled separately using parameters applicable for intrinsically disordered regions using force fields like MARTINI-IDP^73^ or SOP-MULTI,^74^ which combines SOP-SC^55^ and SOP-IDP.^75^ Moreover, for the SOP-SC used here, the single-bead representation of each side chain also does not encode detailed geometry-dependent contacts (e.g., edge-to-edge vs edge-to-face for aromatic–aromatic interactions).^76^ Finally, because the energy function relies on generic native-contact and excluded-volume terms with implicit solvent, it is best suited for describing global mechanical trends from the native state—as used here—rather than providing quantitative residue-level energetics. So all-atom simulations and/or experiments are ultimately required to validate specific interaction hypotheses. For example, we expect that future all-atom simulations to provide atomic-scale insights for several HCM-causing mutations that lay on or near the predicted force-propagation pathway (e.g., F834L and F834Y in H_2_),^5^ as well as detail suboptimal force-propagation pathways.^27^ However, the trade-off in going to all-atom steered molecular dynamics are the typically shorter simulation times (∼1 μs) and the resulting much higher loading rates, 4–6 orders of magnitude high than in experiments. As a result, to be useful, such future all-atom simulations will require longer run times to allow for domain-scale ankle opening before local helical unwinding. Future hardware advances coupled with longer run times should also help close the current 100-fold gap in pulling speeds between coarse-grained simulations and single-molecule assays pulling at sarcomere-relevant contraction velocities (*v* = 400 nm/s).^41^

### Toward the Physiological Role of CALLI and Ankle Opening

A more complete understanding of the physiological role of CALLI awaits measurements of ankle dynamics under a constant applied force at 37 °C and comparing the 1D force applied here to the more complex force-application geometry in the sarcomere^77,78^ (e.g., force and torque^79^). Estimates of the average isometric force for a single myosin range from 6 pN to 9 pN,^80^ with individual peak isometric events reaching 17 pN based on optical-trapping studies at room temperature.^81^ Rat cardiac muscle fiber experiments report a 60% increase in the isometric force per attached myosin head as the temperature was increased from 2 to 17 °C.^82^ If that trend continues, then forces high than 6–9 pN will be generated at 37 °C. On the other hand, we expect that the ankle-opening force will decrease with increasing temperature and under constant force. How these two trends intersect to affect ankle-opening dynamics awaits further experimentation.

That said, we speculate that ankle opening is the molecular origin of the differing dynamics in EF_N_ and EF_C_ seen in bifunctional fluorescent dye experiments on intact mammalian cardiac muscle fibers.^83^ If correct, we would predict a greater differential motion between EF_N_ and EF_C_ for mutations that lower the ankle-opening force. Notably, if these bifunctional fluorescent dye experiments are probing the same ankle-opening transition measured here, then this transition is not simply governed by a 1-D unfolding trajectory along θ. Rather, it requires quantification of a second angle (φ) that is not resolved in the 1D AFM pulling assay and our 1D-energy landscape analysis. These results would also need to be synthesized with the 2D nature of the force and torque generated by a single myosin motor^79^ and points toward the value of merging single-molecule measurements with simulations. Such analysis will build upon the current understanding of how crossbridge compliance affects the mechanics of muscle contraction, as recently reviewed.^80^

Kampourakis & Irving,^83^ based on the bifunctional fluorescent dye experiments, proposed that the differential motion between EF_N_ and EF_C_ promotes entry into the OFF state, adding a third mechanism for how modulation of the mechanical rupture force of CALLI and, more generally, ankle opening or angle could affect muscle physiology. A potential fourth mechanism, also based on the same bifunctional dye experiments, is flexibility at the ankle could change the effective lever-arm length.^84^ These mechanisms add to our initial working hypotheses that ankle stiffness could modulate crossbridge compliance^12^ and/or affect the propensity of myosin to partition between the ON and OFF state.^11^ For the later mechanism, we speculate that an HCM-causing mutation could destabilize CALLI and thereby the OFF state (Figures 1D and 4B). This destabilization would lead to more myosin molecules in the ON state and thus hypercontractility, a cause of HCM. Intriguingly, CALLI contains two such mutations (E22K^85^ and P95A^86^) while a third (D94A^87^) exhibits the opposite phenotype, dilated cardiomyopathy, highlighting how it is not easy to anticipate how multiple molecular mechanisms may contribute to the clinically observed phenotype. As all these mechanisms revolve around a change in ankle angle orientation or stiffness, we expect that pulling across the LA^RLC^ is the most physiologically relevant of the three presented pulling geometries.

## Conclusions

We developed a high-resolution AFM-based single-molecule force spectroscopy assay to probe the nanomechanics of the human β-cardiac myosin’s RLC complex, complementing prior AFM studies of cardiac myosin-binding protein C.^88^ Different polyprotein constructs allowed us to isolate the application of force at different points within the RLC complex. When we applied force across the LA^RLC^—our best proxy for ankle stiffness—two intermediates in the unfolding pathway were detected. Notably, the first intermediate was identified as the opening of the myosin’s ankle, a force-induced conformational change rather than the more traditional force-induced domain unfolding. There was a significant energetic barrier—estimated to be 11–13 *k*_B_ *T*—to opening the ankle of human β-cardiac myosin, a striated muscle myosin. Hence, it is distinct from scallop smooth muscle myosin, where the ankle is interpreted as a source of cross-bridge compliance due to its two structurally resolved ankle angles (77 and 109°).^12^ Notably, the angular transition state for human β-cardiac myosin lies between these two angles. Full RLC dissociation occurred at a remarkably high force [109 ± 2 pN (mean ± SEM; *N* = 100)], when pulling at a 600 nm/s due to the resiliency of the H_1_:EF_C_ interaction. Simulations detailed residues of N-terminal tail (Met^20^–Gln^22^) participating in CALLI to stabilize the acute ankle angle, an interaction that was experimentally confirmed by removing the RLC’s N-terminal tail.

Looking forward, we expect this study to form the foundation for future investigations into the nanomechanics of the RLC complex. Such studies could include characterizing the effects of phosphorylation on ankle stability^89^ and the stability of the ankle in other myosin 2 family members (e.g., scallop smooth muscle myosin^12^). Measuring the consequences of pathogenic mutations, including ones hypothesized to affect ankle angle orientation (F834L^11^) or within the CALLI interface, should be mechanistically insightful, particularly when paired with a recently developed FRET-based assay that measures the fraction of myosin molecules in the ON and OFF state.^18^ Thus, the work presented here enables a rich set of biomedically informative, structure–function experiments.

## Methods

### Polyprotein Design and Preparation

We used a 120-nm ELP to minimize internal tension within the RLC complex. Our original polyprotein construct applied force across the RLC complex (Figure 2C) based on analogy with pioneering studies of calmodulin binding to one of its cognate ligands, MLCK. This construct had a 2-glycine spacer between the end of the calmodulin and MLCK.^47^ This first-generation RLC polyprotein construct showed no mechanical unfolding signature associated with the unfolding of the RLC complex. When the linker was extended to a 20-nm ELP, a mechanical signature akin to Figure 8G was observed, showing the need for a longer linker. The need to go to a much longer ELP arises when pulling across either the LA^RLC^ or the RLC (Figure 2A,B, respectively). For specificity, we illustrate this issue for the assay applying force across the LA^RLC^. By design, there is an 18-aa spacer between the ybbR tag and the start of the LA^RLC^ to minimize any confounding surface interactions by lifting the complex away from the surface when under force and to make sure the Sfp enzyme, used to conjugate the ybbR tag to the CoA-derivatized surface, is not sterically inhibited from binding to the ybbR tag by an immediately adjacent RLC complex. There is also an additional 9-aa under tension since it is the first serine in the ybbR target sequence (D **S** LEFIASKLA) that is conjugated to the CoA-derivatized, PEG-coated surface. As a result, there is a total of 27 aa under tension between the surface and the start of the LA^RLC^. Under tension, the LA^RLC^ is vertically aligned, which positions the C-terminal structured part of the RLC an additional 2.2 nm away from the surface, since the RLC’s C-terminal EF-hand domain (EF_C_) binds to N-terminal portion of the lever arm (H_1_). As a result, under a force of 30 pN, the total height for C-terminal structured part of the RLC is 8.9 nm above the surface as modeled by WLC elasticity for unstructured polypeptide (*p* = 0.4 nm^90^ and rise per amino acid of 0.365 nm/aa^58^). [Note, there is a slight discrepancy in the naming convention for the 120-nm ELP, as defined in the original publication,^30^ and the calculation that we are doing here. The original publication used 0.4 nm/aa for determining the length of a 300-aa ELP, whereas we use 0.365 nm/aa for our calculations, yielding 109.5 nm.] As illustrated Figure 2D, the ELP must stretch in parallel to accommodate the RLC being lifted away from the surface but still bound to the LA^RLC^. For a 20-nm ELP, this degree of stretching would lead to a force of 8.5 pN when neglecting stretching springs in parallel. This force would be applied to the EF_C_ domain of the RLC, yet the goal of the polyprotein design was to apply force only across the LA^RLC^. With a 120-nm ELP, the nominal internal force from stretching the ELP in parallel drops to just 1.3 pN, an essentially negligible force by single-molecule force-spectroscopy protein-folding standards.^91^ Longer ELPs are not easily accommodated because they could self-aggregate.^92^

We cloned recombinant polyproteins into a pET28 vector containing a C-terminal RGSIDTWV affinity tag^93^ (“C-tag”) using Gibson assembly (New England Biolab’s NEBuilder HiFi DNA Assembly Master Mix). Polyproteins were expressed in BL21­(DE3) *E. coli* (New England Biolabs) and purified by standard gravity-flow affinity chromatography techniques using PDZ^93^ coupled to Sulfolink Coupling Resin (Thermo Fisher). Briefly, cell cultures at OD_600 nm_ = 0.6–0.8 were cold shocked for 20 min on ice before induction with 0.2 M isopropylthio-β-galactoside (IPTG) for 4 h at 37 °C. Cell pellets were lysed with 3 freeze-thaw cycles in Lysis Buffer [50 mM Tris (pH7.5), 50 mM NaCl, 0.2% NP-40, 2 M urea, 1 mg/mL lysozyme, and 5 mM DTT] supplemented with a protease inhibitor cocktail (Roche, #4693132001). Bound columns were washed in 3 column volumes each of Wash Buffer 1 [50 mM Tris (pH 7.5), 150 mM KCl, 350 mM NaCl, 2 mM MgCl_2_, and 5 mM DTT] and Wash Buffer 2 [50 mM Tris (pH 7.5), 150 mM KCl, 2 mM MgCl_2_, and 5 mM DTT]. The polyprotein was eluted using 4 column volumes of Elution Buffer [20 mM HEPES (pH 7.5), 150 mM KCl, 2 mM MgCl_2_, 1 mM Tris­(2-carboxyethyl) phosphine (TCEP)] containing 0.125 mg/mL elution peptide (WETWV; synthesized by Genscript). Fractions containing the polyprotein were dialyzed overnight against Storage Buffer [20 mM HEPES (pH 7.5), 150 mM KCl, 2 mM MgCl_2_, and 1 mM TCEP]. To make the Δ*N* construct with residues 2–22 deleted from the RLC, two primers flanking away from the deleted region were used following the standard protocol of the Q5 Site directed Mutagenesis kit (New England Biolabs). Construct plasmids were deposited with Addgene (https://www.addgene.org).

### Coverslip and Modified Cantilever Preparation

To enhance force precision and stability, we fabricated FIB-modified ultrashort cantilevers [*L* = 10 μm (Olympus and RIBM)], as previously described.^94,95^ Cantilever stiffness was calibrated in air using the thermal method,^96^ while sensitivity (V/nm) was determined in liquid by fitting to a power spectrum of the thermal motion of the cantilever.^38^ To site-specifically attach proteins to the coverslips and cantilevers, we purchased commercial amine-functionalized glass coverslips (PolyAn) and functionalized cantilevers with 3-aminopropyl dimethyl ethoxysilane (Gelest) using existing protocols.^97,98^ The cantilevers and coverslips were PEGylated using TFP-ester–PEG_12_–maleimide and TFP-ester–PEG_36_–maleimide (Vector Laboratories), respectively. We replaced the oft-used NHS ester with TFP ester due to its higher stability, resulting in a lower hydrolysis rate.^99^ Proteins were then enzymatically conjugated to the CoA-terminated PEG using Sfp via ybbR, a short genetic tag.^28^

### AFM Assay and Analysis

We used a commercial AFM (Cypher ES, Asylum Research) featuring a temperature-controlled, fluidic chamber and a custom-built detection module that generates a 3-μm spot size.^38^ All experiments were performed in 20 mM HEPES (pH 7.5), 150 mM KCl, 2 mM MgCl_2_ and 2 mM CaCl_2_ at *T* = 25 °C. It is common to have a large optical-interference artifact in the cantilever detection signal with ultrashort cantilevers (10 × 2 μm^2^), in general, and FIB-modified ultrashort cantilevers (∼4 × 2 μm^2^ reflective area), in particular.^38^ This unwanted signal arises from light from the 3-μm-diameter detection laser overfilling the cantilever and reflecting off of the glass coverslip. This reflected light then interferes with light reflected from the cantilever in the far field on the detector. For FIB-modified ultrashort cantilevers on a flat surface, this optical-interference artifact can be hundreds of picoNewtons.^42^ We used two techniques to effectively minimize this problem. First, we tilted the sample by 8° in the opposite direction to the existing 11° tilt of the detection laser, resulting in a ∼10-fold reduction of the interference signal.^100^ We next subtracted out the remaining optical-interference artifact using a previously developed postprocessing routine^42^ (Figure S16A–D, see Supporting Information for details). This routine yielded a small ≤ 2 pN residual, nonsystematic error over the relevant section of the force-extension curve (0–100 nm) (Figure S16D), demonstrating the effectiveness of this combined process. Each cantilever was individually calibrated with an average stiffness of 19 pN/nm and a response time of 2 μs.

We acquired data using two different schemes, one that fully unfolded each individual molecule once and one where the same individual molecule was repeatedly unfolded multiple times to increase the rate of data acquisition. For both schemes, we first pressed the cantilever gently into the surface for brief period (*F* < 80 pN; *t* < 100 ms) to promote the formation of the cohesin-dockerin linkage.^101^ The cantilever was then retracted a fixed velocity (100, 600, 1000, or 5000 nm/s) to unfold the polyprotein construct. When acquiring multiple curves, the data-acquisition protocol stopped cantilever retraction at *F* = 300 pN, a force sufficient to unfold the marker protein (GB1) but not rupture the tip-polyprotein linkage due to the high mechanical stability of the cohesin-dockerin interaction. The cantilever was then returned to the surface (*F* = 0) for 3 s to promote refolding of GB1 and the RLC complex. Retraction velocities were varied to generate a dynamic force spectra^25^ (*v* = 100–10,000 nm/s). During this repeated cycling (Figure S9), cantilever retraction was stopped at 80 pN and then reversed, leading to the opening of myosin’s ankle but not necessarily full RLC dissociation or the unfolding of the marker protein. The zero-force delay was set to 1 s, sufficient for the reformation of the native state of the RLC complex (Figures S9 and S10). After this cycling was finished, we completely detached the protein from the tip by rupturing the cohesin-dockerin interaction. To screen for single-molecule attachment, we used the known mechanical fingerprint of GB1 or ddFLN4 and the rupture of cohesin-dockerin linkage. Cantilever deflection was digitized at 500 kHz when repeatedly unfolding the same individual molecule or performing a single retraction at 5000 nm/s. For single retractions at lower velocities, the digitization was done at 50 kHz.

To analyze the resulting force-extension curves, we used an improved WLC approximation^102^ for the unstructured polypeptide and incorporated a force-dependent transition in the elasticity of PEG.^103^ The mean initial force drop for the N→O transition versus loading rate was analyzed using the Bell–Evans model^25^ to yield Δ*x* ^‡^ and the zero-force unfolding rate (*k*_0_) using *F*_mp_ = (Δ*x*_/_^‡^ *k*_B_ *T*)^−1^ln­[*r*Δ*x* ^‡^/(*k*_0_ *k*_B_ *T*)], where *r* is the loading rate. For individual records, a local line fit to a force-vs-time plot at the rupture point determined the rupture force and the loading rate. Fitting this formula to the dynamic-force-spectroscopy data yielded Δ*x* ^‡^ = 0.58 ± 0.09 nm (fit ± SD) and *k*_0_ = 0.01 ± 0.016 s^–1^.

### Coarse-Grained Brownian Dynamics Simulations

We performed Brownian dynamics simulations^104^ using the self-organized polymer-side chain (SOP-SC) model.^55,105^ This model is structure-based, where each amino acid is represented by an interaction center at the α-carbon (*C*_α_) and the center of mass of the side chain. The strength of the interactions between the different side chains are defined using the Betancourt-Thirumalai statistical potential.^106^ We implemented the force field for the complex following the work by Mondal et al.^57,107^ The energy of the complex for a conformation with coordinates {***r***} is given by

$$E\left(\{r\}\right)=E^{\mathrm{bonded}}+E_{\mathrm{native}}^{\mathrm{non}-\mathrm{bonded}}+E_{\mathrm{non}-\mathrm{native}}^{\mathrm{non}-\mathrm{bonded}}+E_{\mathrm{electrostatic}}^{\mathrm{non}-\mathrm{bonded}}$$

where *E* ^bonded^ represents the energy of the finite extensible nonlinear elastic (FENE) bonded interactions, *E*_native_^non–bonded^ is the energy of the native interactions that are of the Leonard Jones (LJ) type, *E*_non–native_^non–bonded^ is the energy of the repulsive interactions, and *E*_electrostatic_^non–bonded^ is the energy of the electrostatic interactions, as described by the Debye–Hückel potential.

The starting structure for the isolated RLC complex was generated using AlphaFold 3.^56^ We used *alphafoldserver.com* to generate 40 replicas. The sequence of LA^RLC^ and RLC were inputted as protein chains along with a Mg^2+^ ion. The structure with highest pTM (84) and ipTM (0.85) score was used, where pTM and ipTM represent the accuracy in the structure of the complex and in their interface, respectively. The top seven structures were essentially identical except for regions of low confidence (pLDDT < 50), consisting of the N-terminal tail of the RLC (residues 1–14) and the C-terminal end of the LA^RLC^ (residues 36–37) (Figure S4). The backbone RMSD was 0.19 Å between the simulated structure and the six similarly highly ranked structures for the regions of predicted with confidence to high confidence (pLDDT > 70), which encompasses the whole crystallographically resolved structure for the homologous smooth muscle myosin scallop RLC complex.^12^ We note that we discarded predicted structures that contained a kink near the end of H_2_. These predictions were an artifact due to memorization of training data,^108^ particularly the cryo-EM structure.^17^

To generate more realistic looking force-extension curves using this simulation, we anchored the protein via a virtual WLC spring^45^ that quantitatively mimicked a contour length of 6 nm and a persistence length of 0.4 nm.^90^ Besides generating more realistic looking force-extension curves, using a virtual WLC spring enabled fitting the simulated data with the WLC model for the contour-length analysis that compared the simulated molecular configurations to the experimental intermediates. The spring constant of the pulling spring was 8 pN/nm and the pulling velocity was 40 μm/s. The simulations were run on a Nvidia RTX 3090 GPU using the OPENMM software package.^109^ Nine replicas of the complex were pulled in parallel to maximize GPU utilization and thereby more rapidly generate a sufficient number of trajectories to sample nonobligate intermediates along the unfolding pathway. These concurrent replicas had no interactions between them. The simulated trajectories were analyzed using custom python scripts using the MDAnalysis package.^110,111^ A typical 860-μs simulation with these nine replicas took approximately 180 h on an Intel computer [Xeon­(R) processor]. VMD 2.0 ^112,113^ and open-source PyMOL^114^ were used for visualization.

The fraction of native contacts *Q*_ankle_ that stabilize the native state against ankle opening was used to estimate the transition state. To do so, we first filtered out the contacts from the entire complex that did not persist for 90% of the trajectory segment in the native state from the set of all contacts in the complex. Among these remaining contacts, we again filtered out the contacts that were not broken for 90% of the trajectory segment in the open-ankle state. The angle of the ankle when *Q*_ankle_ crosses 0.5 for the first time in the trajectory was used to estimate the transition state.

## Supplementary Material















## Data Availability

The data that supports the findings of this paper are openly available on Dryad (DOI: 10.5061/dryad.mpg4f4rg9).

## Notes

Additional material and methods along with figures and tables, including a diagram and table of pathogenic mutations in the LA^RLC^, quantification of the physical properties of FIB modified ultrashort and uncoated BioLever Long cantilevers, alignment of top structures predicted by AlphaFold 3, a diagram of the data-acquisition process, methodology for deducing the location of the rotational transition state, ribbon diagrams of CALLI in the OFF state, the full amino acid sequences of the polyprotein constructs, and the pairwise interactions stabilizing CALLI (PDF)
Movie 1: Pulling nine RLC complexes in parallel (MP4)
Movie 2: The unfolding of LA^RLC^ along with dissociation of RLC, when pulling across LA^RLC^ (MP4)
Movie 3: Back and forth transitions between the native and the open state (MP4)
Movie 4: Tethered Brownian motion of EF_N_ in the I_2_ state with transient reformation of CALLI (MP4)
Movie 5: Two unfolding pathways when pulling across RLC (MP4)
Movie 6: The unfolding pathway when pulling across the RLC complex (MP4)
