# Supplementary material for: Force-Induced Ankle Opening Reveals Mechanical Stabilization of the Ankle of Human β‑Cardiac Myosin
Source: ACS Nano. 2026 May 21;20(22):15982–6000. doi: 10.1021/acsnano.5c22720 (PMC13255530; doi:10.1021/acsnano.5c22720)
Supplement: Supplementary file 1 [file nn5c22720_si_001.pdf]

Supporting Information for

Force-Induced Ankle Opening Reveals Mechanical Stabilization of the Ankle of Human β-Cardiac Myosin

Surya Pratap S. Deopa,[1] Kristen K. Bjorkman,[2,3] Devin T. Edwards,[1] Marc-André LeBlanc,[1] Massimo Buvoli,[2,3] Steven H. Oakes,[1,2,4] Brandon Dzuba,[1] Anastasia Karabina,[2,3] Leslie A. Leinwand,*[,2,3] and Thomas T. Perkins,*[,1,2]

[1]JILA, National Institute of Standards and Technology and University of Colorado, Boulder, Colorado 80309, United States

[2]Department of Molecular, Cellular, and Developmental Biology, University of Colorado, Boulder, Colorado 80309, United States

[3]BioFrontiers Institute, University of Colorado, Boulder, Colorado 80303, United States

[4]Department of Physics, University of Colorado, Boulder, Colorado 80309, United States

*Corresponding emails: leslie.leinwand@colorado.edu, tperkins@colorado.edu

**Materials and Methods**	S3–4
**Polyprotein sequences**	S5–6
**Figures:**	
Figure S1	S7
Figure S2	S8
Figure S3	S9
Figure S4	S10
Figure S5	S11
Figure S6	S12
Figure S7	S13
Figure S8	S14
Figure S9	S15
Figure S10	S16
Figure S11	S17
Figure S12	S18
Figure S13	S19
Figure S14	S20
Figure S15	S21
Figure S16	S21
**Tables:**	
Table S1	S22
Table S2	S23
Table S3	S23
Table S4	S24
**References**	S25

Material and methods

Cantilever calibration. Hard contact on a tilted sapphire glass surface in air was used to determine the sensitivity (nm/V) of the detector. This hard contact was repeated ten times and averaged for the final value. Next, the cantilever was retracted away from the surface (~3 µm), and power spectral density (PSD) measurements were performed. The simple harmonic model was used to fit the PSD to obtain cantilever stiffness.[1] This measurement of cantilever stiffness was done at a maximum and a minimum of optical-interference artifact and then averaged (Figure S16).

Coverslip and cantilever functionalization. Cantilevers were ozone plasma treated for 15 min and then immersed in a solution consisting of 1 mL of aminopropyl dimethyl ethoxysilane (Gelest), 1 mL ethanol, and 5 µl water for 15 min. The cantilevers were then rinsed in ethanol and water for 1 min each. Next, we baked the cantilevers in a vacuum oven for 30 min at 80 °C. For the coverslips, we started with commercially available amine-functionalized coverslips (PolyAn) that were scribed into small squares (~3×3 mm^2) for use in the AFM.

The amine-functionalized cantilevers and coverslips were immersed in a 50 mM borate buffer (pH 8.5) for 1 hr. We prepared solutions of 20 mM tetrafluorophenyl esters (TFP)-PEG_{36}-maleimide (Vector labs) and TFP-PEG_{12}-maleimide in 50 mM borate buffer (pH 8). Each solution was spun at 15 krpm for 10 min. We then pipetted off the supernatant of the PEG_{36} and PEG_{12} solutions and applied it directly onto the coverslip and cantilevers, respectively. After 1 h, the coverslips and cantilevers were rinsed thoroughly. To functionalize with coenzyme A (CoA), we applied 1 mM CoA in 50 mM sodium phosphate (pH 7.5), 50 mM NaCl, 10 mM EDTA, and 2 mM TCEP to the coverslips and the cantilevers for 1 h. The CoA-functionalized cantilevers could be stored at 4 °C for a week in liquid. The coverslips were rinsed, dried using N_2, and stored under N_2 at 4 °C and could be used for up to two months.

Site-specific protein conjugation. We used Sfp to enzymatically catalyze the coupling of the ybbR tag[2] to the CoA-functionalized surfaces for both the polyprotein to the coverslips and cohesin to the cantilevers based around previously developed proptocols.[3] To do so, we prepared 0.5 µM of target protein in Coupling Buffer [20 mM HEPES (pH 7.5), 150 mM KCl, 5 µM Sfp and 20 mM $MgCl_2$]. We first rinsed the coverslips and cantilevers with water and then pipetted on the Coupling Buffer onto the CoA-functionalized and let sit at room temperature for 1 h, after which they were rinsed with at least 100x volume of Experiment Buffer [20 mM HEPES (pH 7.5), 150 mM KCl, 2 mM $MgCl_2$, 2mM $CaCl_2$].

Optical-interference artifact. To subtract out the interferometric-like fringes, we used a previously developed phenomenological formula[4].

$$\Delta z_{\text{Interference}} = w_1 + w_2 x + (w_3 + w_4 x)\sin\big((w_5 + w_6 x)x + w_7\big) \tag{1}$$

$$x = Z_{\text{PZT}} - \Delta z_{\text{Cantilever}} \tag{2}$$

$$\Delta z_{\text{Cantilever}} = \Delta z_{\text{Measured}} - \Delta z_{\text{Interference}} \tag{3}$$

where $\Delta z_{\text{Measured}}$ is the raw measured cantilever deflection which includes the optical-interference artifact. $\Delta z_{\text{Interference}}$ is the contribution of the artifact to $\Delta z_{\text{Measured}}$ and $\Delta z_{\text{Cantilever}}$ is the actual deflection of the cantilever with which the force on the cantilever can be calculated. Z_{PZT} is the distance the cantilever is retracted and x is the extension (tip-sample separation). $w_1 - w_7$ are fitting parameters. During approach before contact, and after protein detachment in the retraction curve $\Delta z_{\text{Cantilever}} = 0$. We concatenated these regions of the approach and retraction curves, and fit them with Eq 1, with $x = Z_{\text{PZT}}$ to obtain $w_1 - w_7$ (Figure S16). Using these parameters, roots of the Eq 3 were found numerically to obtain $\Delta z_{\text{Cantilever}}$ using IGOR Pro 9's inbuilt root finding algorithm taking $\Delta z_{\text{Measured}}$ as the initial guess. This root-finding process was repeated for each point in the force-extension curve.

Force-propagation pathway. The force-propagation pathway analysis used only the backbone-backbone interactions, based on the pioneering all-atom analysis that used only C_α atom correlations.[5] To do so, we first selected for native backbone-backbone contacts that persisted for more than 75% of the selected trajectory range. Next, the correlation coefficients between these backbone interactions were found using a previously developed formulism.[6]

$$\boldsymbol{C_{ij}} = \frac{\text{Cov}_{\boldsymbol{ij}}}{\left(\langle \Delta\vec{r_i}(t)^2\rangle\langle \Delta\vec{r_j}(t)^2\rangle\right)^{\frac{1}{2}}} \tag{4}$$

where $\text{Cov}_{ij} = \langle \Delta\vec{r_i}(t) \cdot \Delta\vec{r_j}(t)\rangle$ and $\Delta\vec{r_i}(t) = \vec{r_i}(t) - \langle\vec{r_i}(t)\rangle$. $\vec{r_i}(t)$ denotes the position of the ith backbone bead at time t and $\langle\ \rangle$ denotes the average. The cross correlation C_{ij} between these backbone beads was calculated with custom python scripts utilizing the MDAnalysis python library. From the correlation coefficient matrix, a network graph was created whose nodes represented the interaction centers and the edge distances between them were given by $w_{ij} = -\log\big(|C_{ij}|\big)$. The optimum force-propagation pathway between the terminals of the LA^{RLC} was obtained by finding the shortest path between them in this graph, using the Floyd-Warshall algorithm implemented in ngxgraph python library. Visualization of the pathway was done using open-source PyMol.[7]

Polyprotein sequences:

Pulling across LA^{RLC}: RLC-ELP120-TEV-ybbR-Lever arm-GB1-GB1-Xmod-DocIII-Ctag

MAPKKAKKRAGGANSNVFSMFEQTQIQEFKEAFTIMDQNRDGFIDKNDLRDTFAALGRVNVKNE
EIDEMIKEAPGPINFTVFLTMFGEKLKGADPEETILNAFKVFDPEGKGVLKADYVREMLTTQAE
RFSKEEVDQMFAAFPPDVTGNLDYKNLVHIITHGEEKDSTVPGEGVPGVGVPGVGVPGVGVPGV
GVPGAGVPGAGVPGGGVPGGGVPGEGVPGEGVPGVGVPGVGVPGVGVPGVGVPGAGVPGAGVPG
GGVPGGGVPGEGVPGEGVPGVGVPGVGVPGVGVPGVGVPGAGVPGAGVPGGGVPGGGVPGEGVP
GEGVPGVGVPGVGVPGVGVPGVGVPGAGVPGAGVPGGGVPGGGVPGEGVPGEGVPGVGVPGVGV
PGVGVPGVGVPGAGVPGAGVPGGGVPGGGVPGEGVPGEGVPGVGVPGVGVPGVGVPGVGVPGAG
VPGAGVPGGGVPGGGVPGEGELENLYFQSDVDSLEFIASKLAGSGSGSGSAGTGSGDIASSLLV
IQWNIRAFMGVKNWPWMKLYFKIKPLLKSAEREGRSGTGSDTYKLILNGKTLKGETTTEAVDAA
TAEKVFKQYANDNGVDGEWTYDDATKTFTVTERSDTYKLILNGKTLKGETTTEAVDAATAEKVF
KQYANDNGVDGEWTYDDATKTFTVTEGSGSDVGNTVTSAVKTQYVEIESVDGFYFNTEDKFDTA
QIKKAVLHTVYNEGYTGDDGVAVVLREYESEPVDITAELTFGDATPANTYKAVENKFDYEIPVY
YNNATLKDAEGNDATVTVYIGLKGDTDLNNIVDGRDATATLTYYAATSTDGKDATTVALSPSTL
VGGNPESVYDDFSAFLSDVKVDAGKELTRFAKKAERLIDGRDASSILTFYTKSSVDQYKDMAAN
EPNKLWDIVTGDAEEETSRGSIDTWV

Pulling across ΔN-LA^{RLC}: RLC(Δ2-22)-ELP120-TEV-YbbR-Lever arm-GB1-GB1-Xmod-DocIII-Ctag

MQTQIQEFKEAFTIMDQNRDGFIDKNDLRDTFAALGRVNVKNEEIDEMIKEAPGPINFTVFLTM
FGEKLKGADPEETILNAFKVFDPEGKGVLKADYVREMLTTQAERFSKEEVDQMFAAFPPDVTGN
LDYKNLVHIITHGEEKDSTVPGEGVPGVGVPGVGVPGVGVPGVGVPGAGVPGAGVPGGGVPGGG
VPGEGVPGEGVPGVGVPGVGVPGVGVPGVGVPGAGVPGAGVPGGGVPGGGVPGEGVPGEGVPGV
GVPGVGVPGVGVPGVGVPGAGVPGAGVPGGGVPGGGVPGEGVPGEGVPGVGVPGVGVPGVGVPG
VGVPGAGVPGAGVPGGGVPGGGVPGEGVPGEGVPGVGVPGVGVPGVGVPGVGVPGAGVPGAGVP
GGGVPGGGVPGEGVPGEGVPGVGVPGVGVPGVGVPGVGVPGAGVPGAGVPGGGVPGGGVPGEGE
LENLYFQSDVDSLEFIASKLAGSGSGSGSAGTGSGDIASSLLVIQWNIRAFMGVKNWPWMKLYF
KIKPLLKSAEREGRSGTGSDTYKLILNGKTLKGETTTEAVDAATAEKVFKQYANDNGVDGEWTY
DDATKTFTVTERSDTYKLILNGKTLKGETTTEAVDAATAEKVFKQYANDNGVDGEWTYDDATKT
FTVTEGSGSDVGNTVTSAVKTQYVEIESVDGFYFNTEDKFDTAQIKKAVLHTVYNEGYTGDDGV
AVVLREYESEPVDITAELTFGDATPANTYKAVENKFDYEIPVYYNNATLKDAEGNDATVTVYIG
LKGDTDLNNIVDGRDATATLTYYAATSTDGKDATTVALSPSTLVGGNPESVYDDFSAFLSDVKV
DAGKELTRFAKKAERLIDGRDASSILTFYTKSSVDQYKDMAANEPNKLWDIVTGDAEEETSRGS
IDTWV

Pulling across RLC: Lever arm-ELP120-TEV-ybbR-RLC-ddFLN4-Xmod-DocIII-Ctag

MSLLVIQWNIRAFMGVKNWPWMKLYFKIKPLLKSAERESTVPGEGVPGVGVPGVGVPGVGVPGV
GVPGAGVPGAGVPGGGVPGGGVPGEGVPGEGVPGVGVPGVGVPGVGVPGVGVPGAGVPGAGVPG
GGVPGGGVPGEGVPGEGVPGVGVPGVGVPGVGVPGVGVPGAGVPGAGVPGGGVPGGGVPGEGVP
GEGVPGVGVPGVGVPGVGVPGVGVPGAGVPGAGVPGGGVPGGGVPGEGVPGEGVPGVGVPGVGV
PGVGVPGVGVPGAGVPGAGVPGGGVPGGGVPGEGVPGEGVPGVGVPGVGVPGVGVPGVGVPGAG
VPGAGVPGGGVPGGGVPGEGELENLYFQSDVDSLEFIASKLAGSGSGSGSAGTGSGDIASMAPK
KAKKRAGGANSNVFSMFEQTQIQEFKEAFTIMDQNRDGFIDKNDLRDTFAALGRVNVKNEEIDE
MIKEAPGPINFTVFLTMFGEKLKGADPEETILNAFKVFDPEGKGVLKADYVREMLTTQAERFSK
EEVDQMFAAFPPDVTGNLDYKNLVHIITHGEEKDGRSGTGSADPEKSYAEGPGLDGGESFQPSK
FKIHAVDPDGVHRTDGGDGFVVTIEGPAPVDPVMVDNGDGTYDVEFEPKEAGDYVINLTLDGDN
VNGFPKTVTVKPAPGSGSGTGNTVTSAVKTQYVEIESVDGFYFNTEDKFDTAQIKKAVLHTVYN
EGYTGDDGVAVVLREYESEPVDITAELTFGDATPANTYKAVENKFDYEIPVYYNNATLKDAEGN
DATVTVYIGLKGDTDLNNIVDGRDATATLTYYAATSTDGKDATTVALSPSTLVGGNPESVYDDF
SAFLSDVKVDAGKELTRFAKKAERLIDGRDASSILTFYTKSSVDQYKDMAANEPNKLWDIVTGD
AEEETSRGSIDTWV

Pulling across the complex: ybbR-ddFLN4-RLC-ELP120-Lever arm-Xmod-DocIII-Ctag

MDSLEFIASKLAGSGSGSGSAGTGSGDIASGSADPEKSYAEGPGLDGGESFQPSKFKIHAVDPD
GVHRTDGGDGFVVTIEGPAPVDPVMVDNGDGTYDVEFEPKEAGDYVINLTLDGDNVNGFPKTVT
VKPAPGSGSSTMAPKKAKKRAGGANSNVFSMFEQTQIQEFKEAFTIMDQNRDGFIDKNDLRDTF
AALGRVNVKNEEIDEMIKEAPGPINFTVFLTMFGEKLKGADPEETILNAFKVFDPEGKGVLKAD
YVREMLTTQAERFSKEEVDQMFAAFPPDVTGNLDYKNLVHIITHGEEKDGGRSVPGEGVPGVGV
PGVGVPGVGVPGVGVPGAGVPGAGVPGGGVPGGGVPGEGVPGEGVPGVGVPGVGVPGVGVPGVG
VPGAGVPGAGVPGGGVPGGGVPGEGVPGEGVPGVGVPGVGVPGVGVPGVGVPGAGVPGAGVPGG
GVPGGGVPGEGVPGEGVPGVGVPGVGVPGVGVPGVGVPGAGVPGAGVPGGGVPGGGVPGEGVPG
EGVPGVGVPGVGVPGVGVPGVGVPGAGVPGAGVPGGGVPGGGVPGEGVPGEGVPGVGVPGVGVP
GVGVPGVGVPGAGVPGAGVPGGGVPGGGVPGEGGTSLLVIQWNIRAFMGVKNWPWMKLYFKIKP
LLKSAEREELRSGDNTVTSAVKTQYVEIESVDGFYFNTEDKFDTAQIKKAVLHTVYNEGYTGDD
GVAVVLREYESEPVDITAELTFGDATPANTYKAVENKFDYEIPVYYNNATLKDAEGNDATVTVY
IGLKGDTDLNNIVDGRDATATLTYYAATSTDGKDATTVALSPSTLVGGNPESVYDDFSAFLSDV
KVDAGKELTRFAKKAERLIDGRDASSILTFYTKSSVDQYKDMAANEPNKLWDIVTGDAEEETSR
GSIDTWV

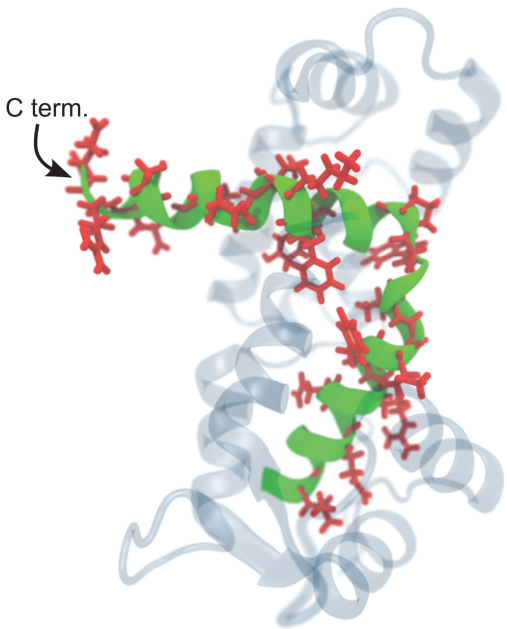

Figure S1. Locations of pathogenic mutations within the LA^{RLC}. Ribbon diagram of the AlphaFold 3 predicted structure of the RLC complex where the LA^{RLC} is shown in green and the RLC in grey. The locations where pathogenic mutations[8] map to are indicated by highlighting their native amino-acid structure in red.

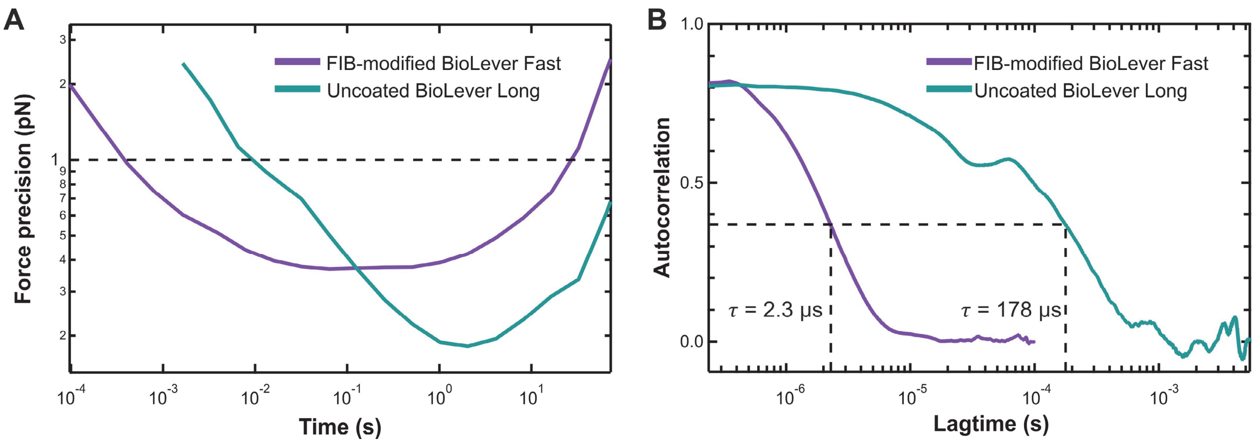

Figure S2. Characterization of FIB-modified ultrashort BioLever Fast and uncoated BioLever Long AFM cantilevers in liquid at 100 nm above the surface determined from its thermal motion. (**A**) Force precision plotted as a function of averaging time. The FIB-modified cantilever (purple) shows sub-pN force precision over essentially 5 decades of time (0.4 ms to 30 s) and reaches sub-pN force precision ~25 fold faster than uncoated BioLever Long (cyan). (**B**) Autocorrelation of the cantilever's thermal motion yields the cantilever response time[9] when the autocorrelation drops by e^{-1}. The FIB-modified BioLever Fast (purple) had an ~80-fold faster response time compared to uncoated BioLever Long (cyan) (τ = 2.3 μs vs 178 μs).

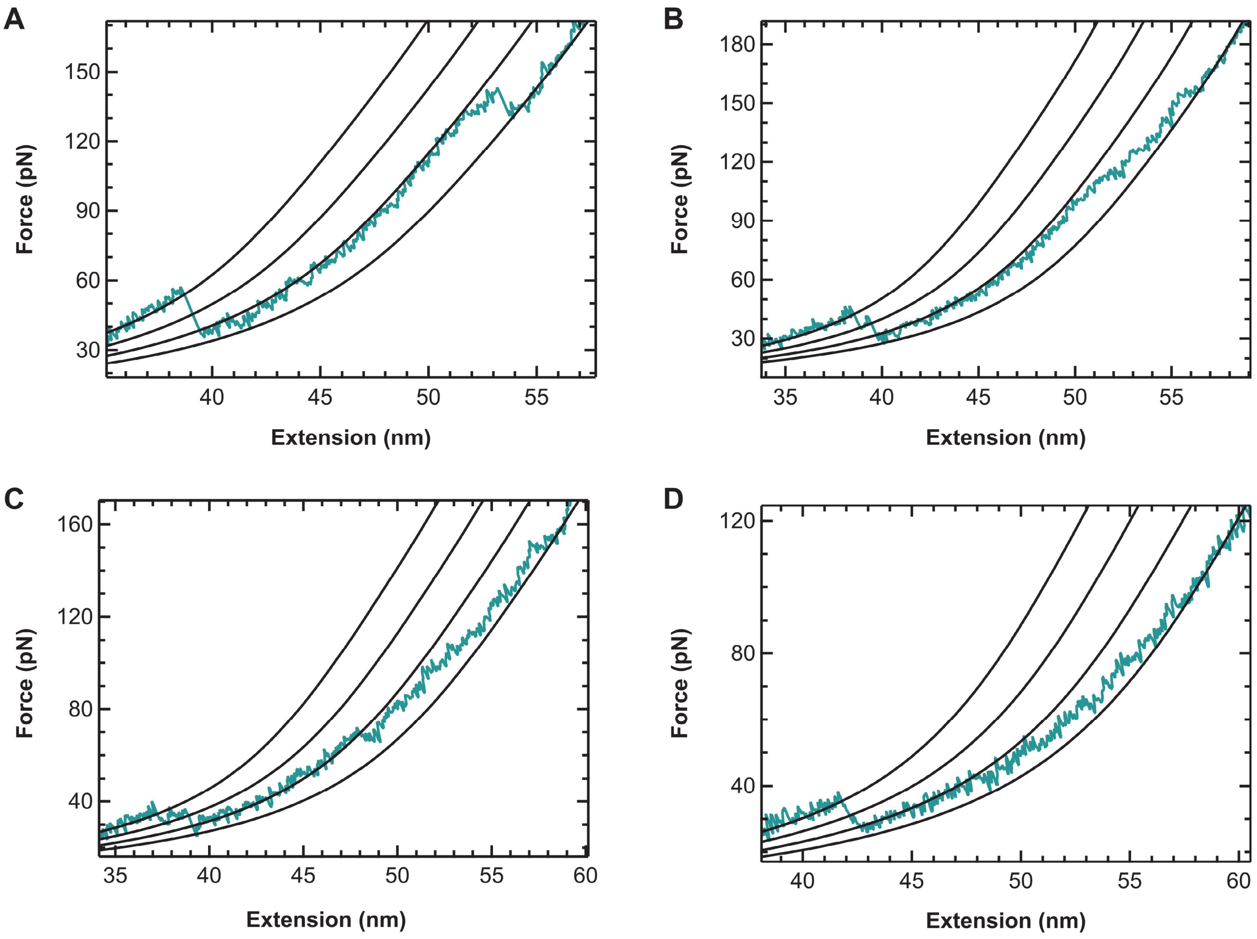

Figure S3. Varied unfolding trajectories. (**A**) A force-extension plot showing a trajectory where no first intermediate was observed (**B–D**) Force-extension curves that show no discrete transition from the second intermediate but rather a gradual transition. The computationally predicted worm-like chain curves, as shown in Figure 4D,G are added to guide the eye and correspond to the four predicted states [native (N), open (O), I_2, and unfolded (U)], where the curve for the second intermediate is its initial contour length. The computationally predicted ΔL_o for the three transitions are N→ O [2.63 ± 0.06 nm (fit ± SD)], O→I_2 (2.78 ± 0.03), and I_2 →U (2.88 ± 0.02). The computationally predicted H_2 helix unraveling is the molecular mechanism by which the I_2 state goes from the second intermediate to the fully unfolded state, as discussed in the main text and Figure 5B.

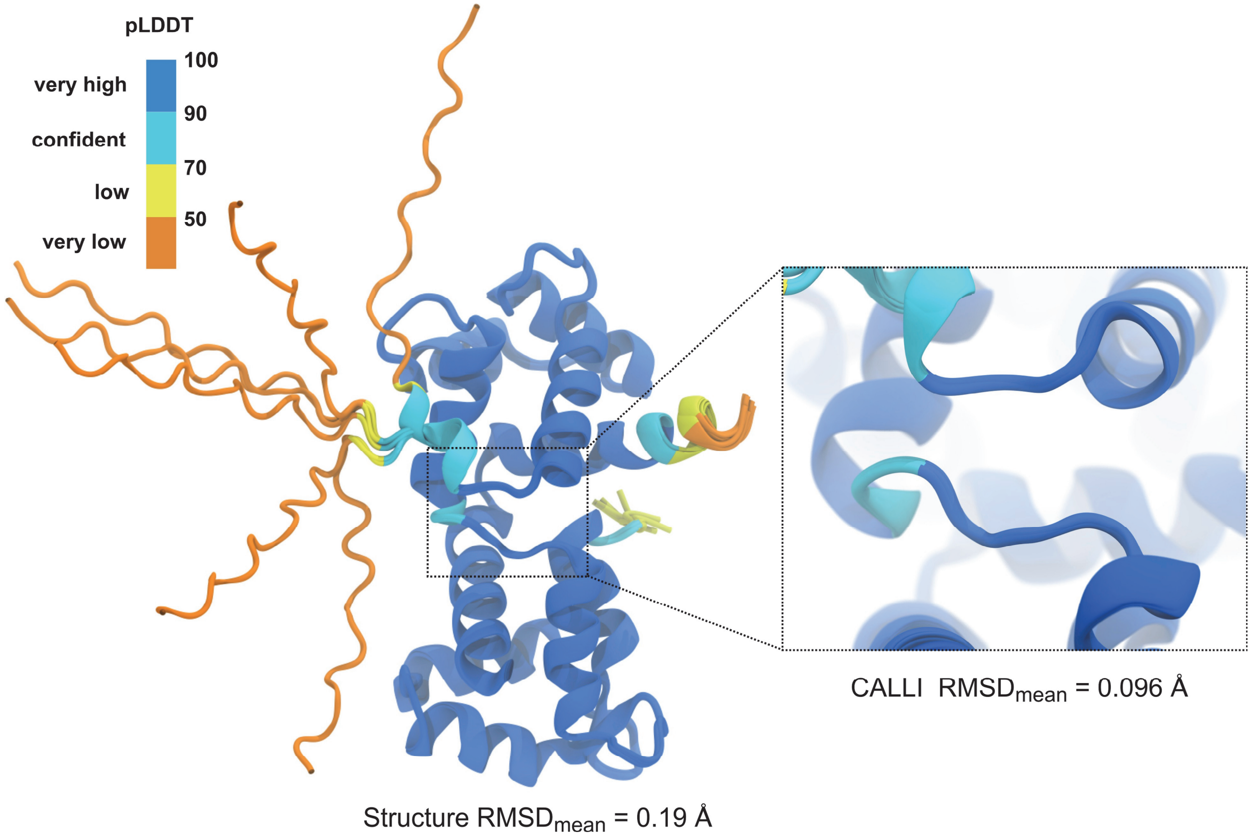

Figure S4. Alignment and RMSD of the top seven AlphaFold 3 predicted structures. (**Left**) Ribbon diagram of our selected starting structure for the simulation (ipTM = 0.85, pTM = 0.85) superimposed with the ribbon diagrams of six other top-ranked structures (ipTM=0.85, pTM=0.84–0.85) aligned to the starting structure using the backbone atoms of residues that with have pLDDT > 70. Note, pTM and ipTM represent the accuracy in the structure of the complex and in their interface, respectively while pLDDT is the per residue measure of local confidence. The mean RMSD of the backbone atoms of the six other structures from the structure used for the simulations is shown for residues with pLDDT > 70. Note, the location of the unstructured N-terminal tail of the RLC is predicted with very low confidence. The last 5 acids in the C-terminal tail of the LA^{RLC} are also predicted with low to very low confidence. (Inset) Ribbon diagrams of the CALLI interface for the seven structures show no discernible deviation in the structures, as evidenced by the even smaller RMSD when aligned to and computed for the residues within CALLI.

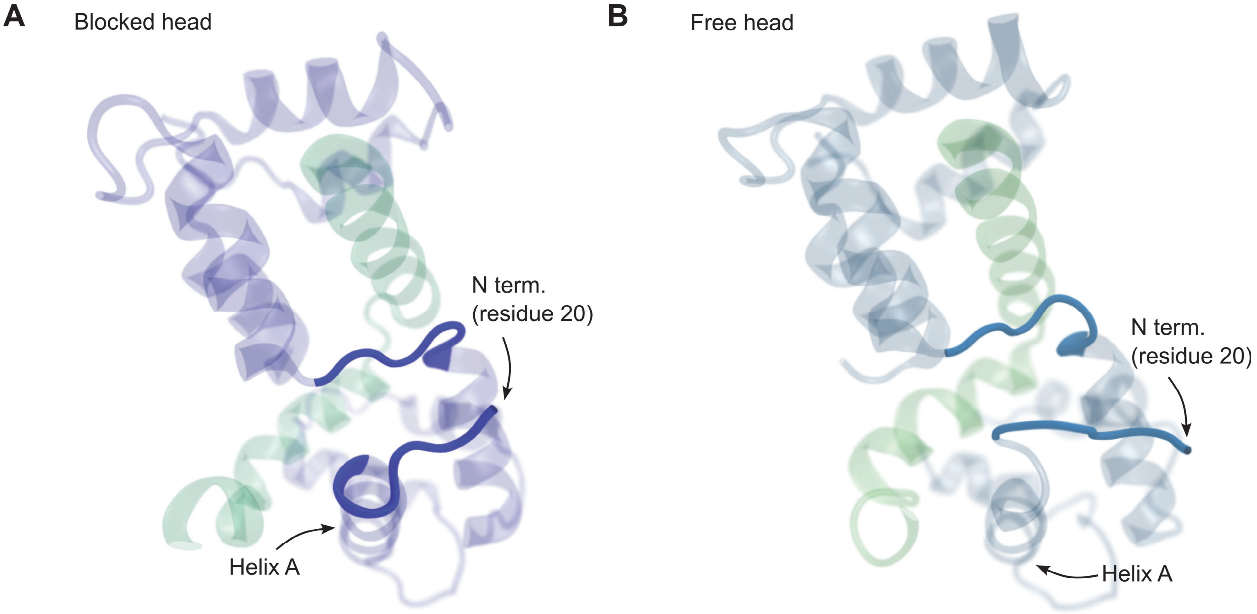

Figure S5. CALLI and the RLC complex in the OFF state of human β-cardiac myosin as resolved by cryoEM (PDB:8ACT).[10] The first 19 amino acids of the unstructured RLC N terminus were unresolved. (**A**) A ribbon diagram of the blocked head shows that CALLI (dark blue) is maintained. (**B**) For the free head, a ribbon diagram shows CALLI is distorted.

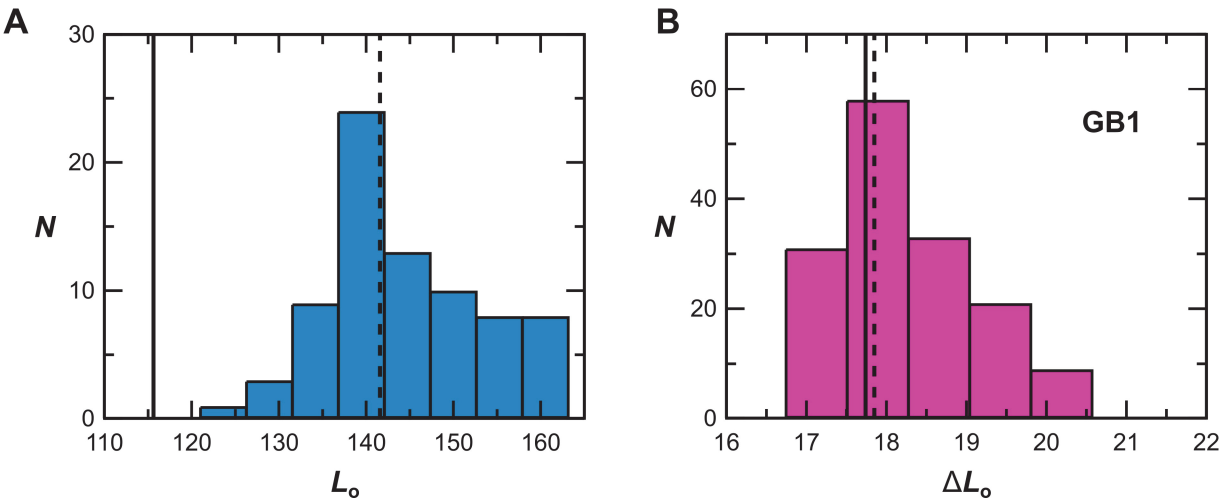

Figure S6. Histogram of absolute total contour length (L_o) of the construct and change in contour length (ΔL_o) due to GB1 unfolding. (**A**) Average L_o [142 ± 1 nm (mean ±SEM), N = 76] and theoretical L_o (116 nm) depicted by dashed and solid vertical lines, respectively. The theoretical L_o is calculated using 48 PEG monomers per the manufacturer's specifications. The deviation in experimentally measured L_o from theoretical value primarily arises from polydispersity in PEG molecules. (**B**) Average ΔL_o [17.85 ± 0.07 nm (mean ± SEM; N = 152)] and theoretical ΔL_o (17.74 nm) due to GB1 unfolding represented by dashed and solid vertical lines respectively. Theoretical ΔL_o is calculated using 0.365 nm[11] as the ΔL_o per amino acid and matches previously reported ΔL_o values for GB1 [18.0 ±0.5 (mean ± SD; N = 472)],[12] showing accurate determination of ΔL_o despite variations in L_o arising from the PEG polydispersity.

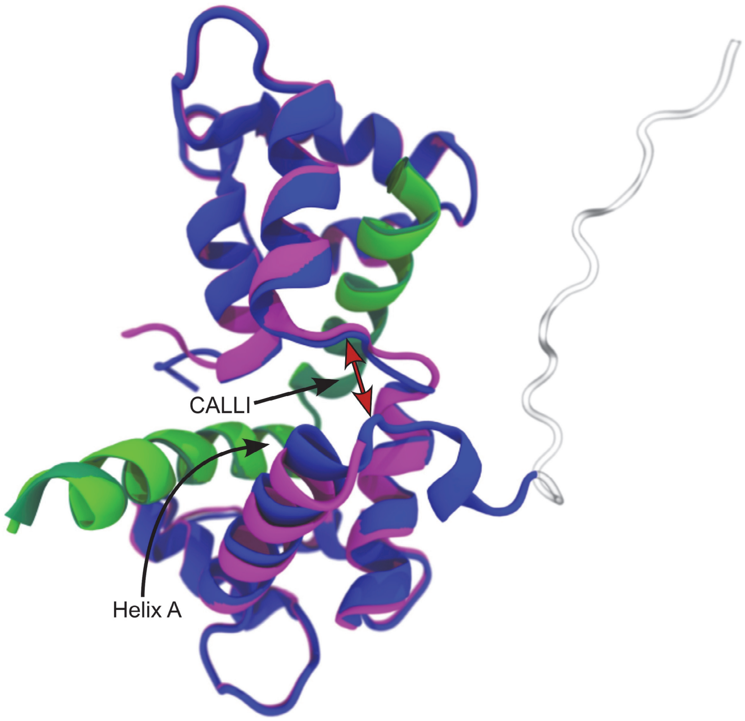

Figure S7. Superposition of the ribbon diagrams of the ΔN and WT constructs, as predicted by AlphaFold 3, where ΔN constrict is color coded dark green (LA^{RLC}) and magenta (RLC). The WT structure is color coded light green (LA^{RLC}) and blue (RLC). Backbone atoms in LA^{RLC} were used for the alignment. Structured regions of ΔN deviate slightly from WT at the start of helix A. The N-terminal tail of the WT RLC predicted with pLDDT < 50 is shown in light grey.

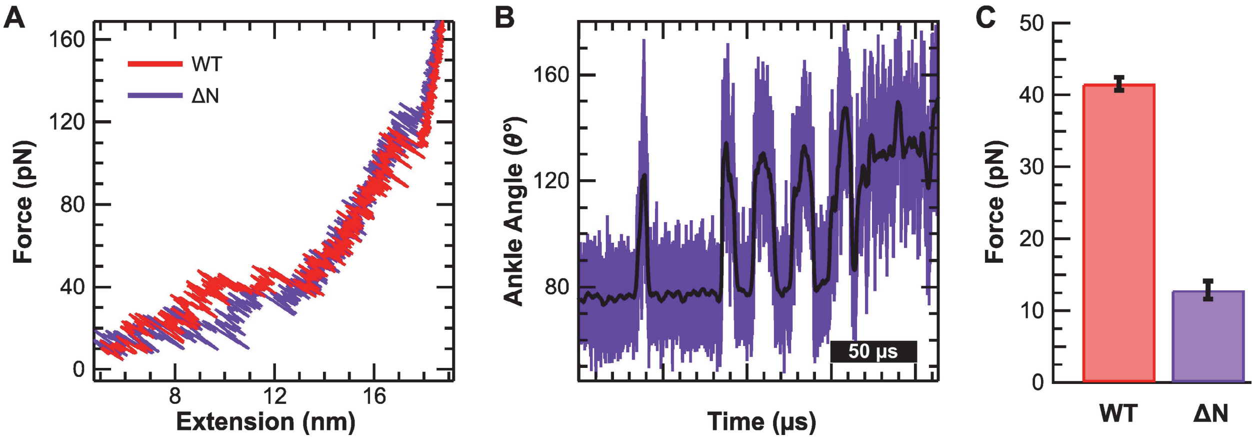

Figure S8. Coarse-grained Brownian dynamics simulations of the ΔN construct. (**A**) Representative simulated force-extension curves show the ΔN construct undergoing an N→O transition at a lower force than the WT construct (purple and red, respectively). (**B**) Representative back-and-forth transitions between the N and O states in the ΔN-construct simulation. (**C**) A bar graph shows the computationally predicted initial ankle-opening force for WT [41.5 ± 0.9 pN (mean ± SEM), $N = 10$] and ΔN (12.9 ± 1.3 pN, $N = 18$) constructs.

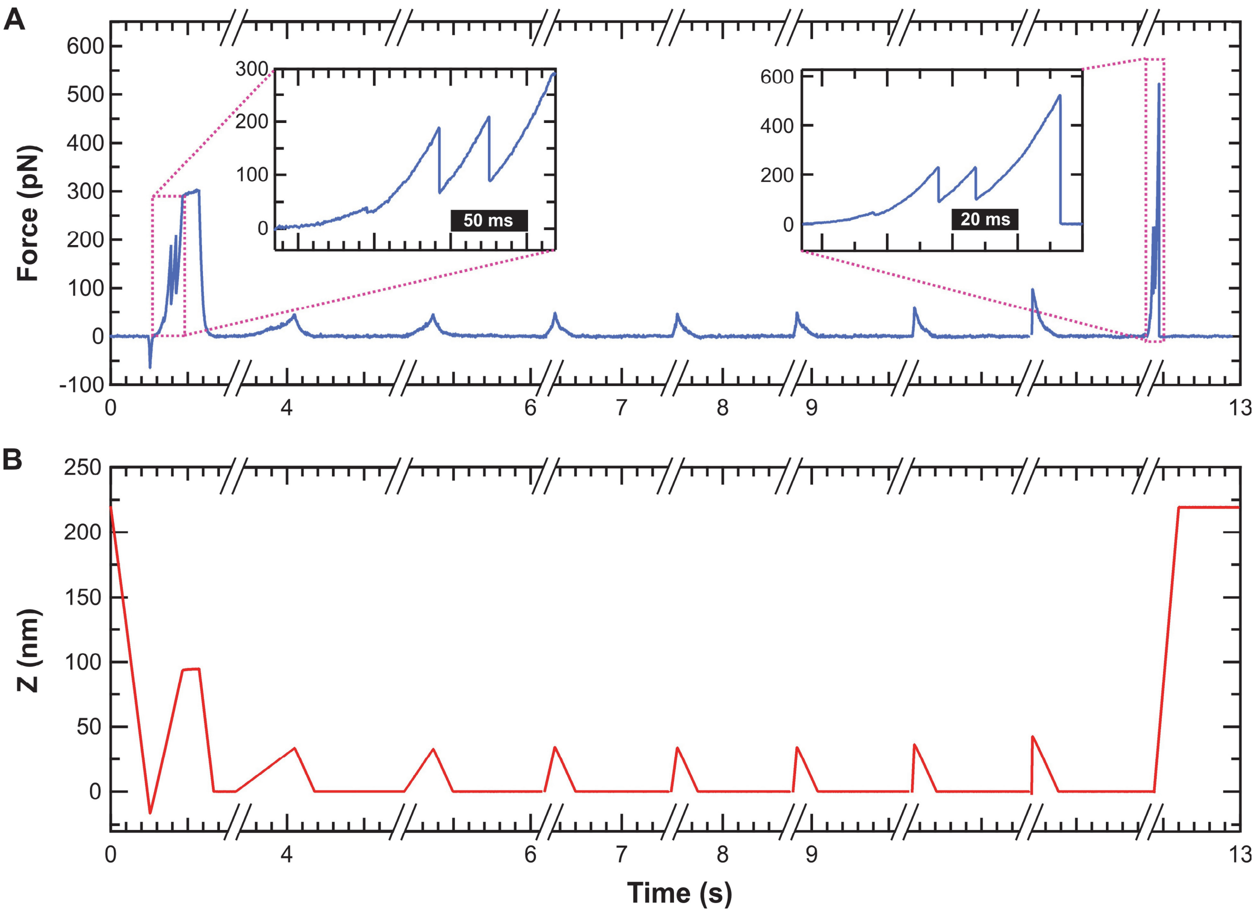

Figure S9. Data-acquisition protocol for the dynamic force spectrum. (**A**) Force plotted as a function of time. After the initial approach and dwell on the surface for protein attachment, the cantilever was retracted with a trigger force set to 300 pN. If this force was achieved, the retraction was halted, and the cantilever was ramped back to the surface. After dwelling for 3 s at zero force for the GB1 domains to fold, a set of reatration-approach cycles was initiated. The retraction velocity was increased after every cycle separated by 1 s dwell to provide refolding time for the RLC complex. The set of velocities studied was 100, 200, 600, 1000, 1600, 3000 and 10000 nm/s with an approach velocity of 300 nm/s. After this cycling, the cantilever was retracted fully to detach the protein from the cantilever. Single-molecule attachments were confirmed by looking for the mechanical signature of GB1 unfolding after the initial attachment (left inset) and final detachment (right inset). (**B**) Cantilever position (*Z*) plotted as a function of time. The breaks in time axis represent a portion of the zero force dwells where data were not recorded.

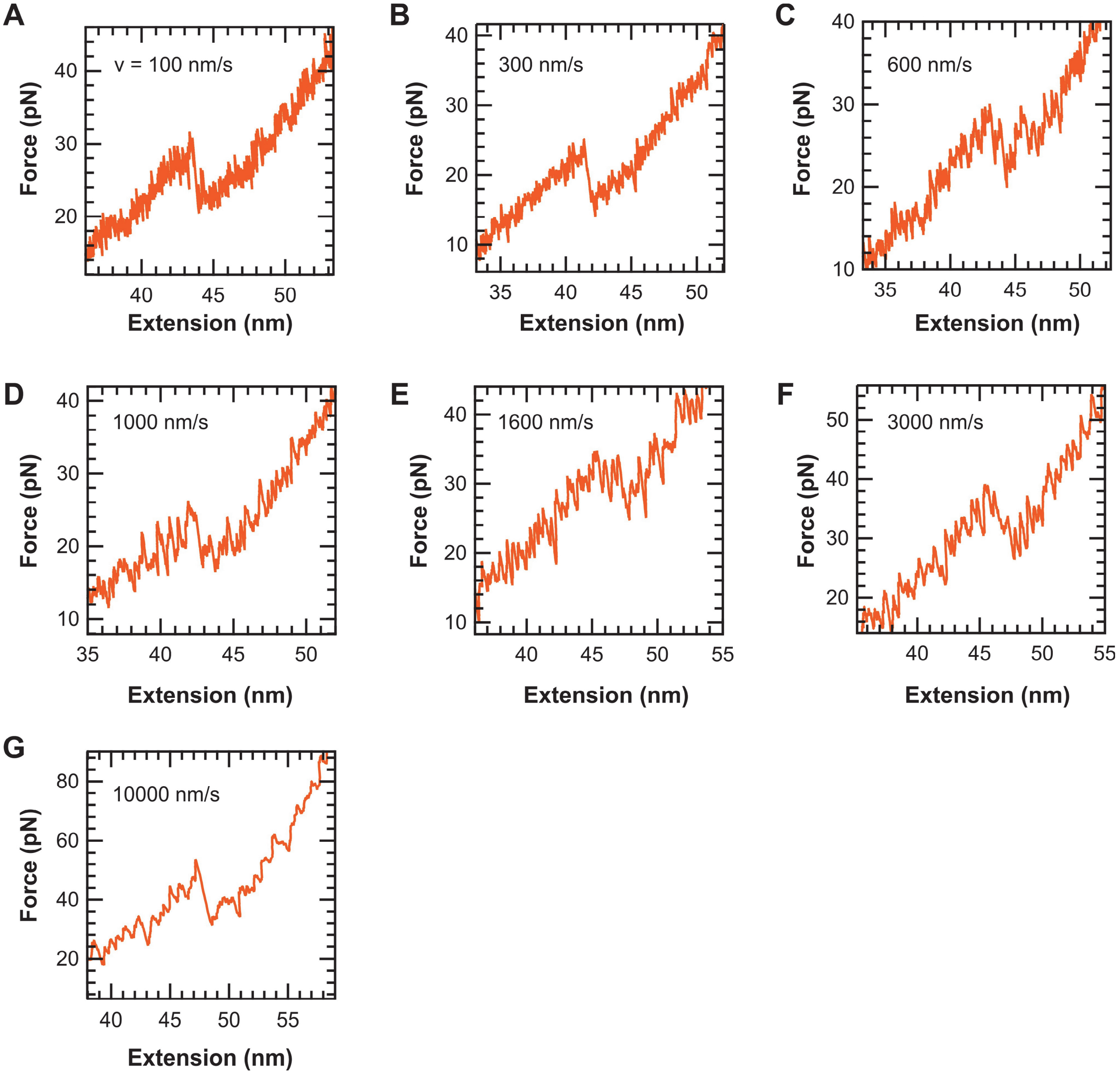

Figure S10. Seven consecutive force-extension curves show the expected mechanical fingerprint of a fully folded RLC complex when pulling across the LA^{RLC} when using the pulling protocol depicted in Figure S9 that has a 1-s dwell between the stretching cycles, showing that 1 s was sufficient for the reformation of the native RLC complex after stretching to 80 pN.

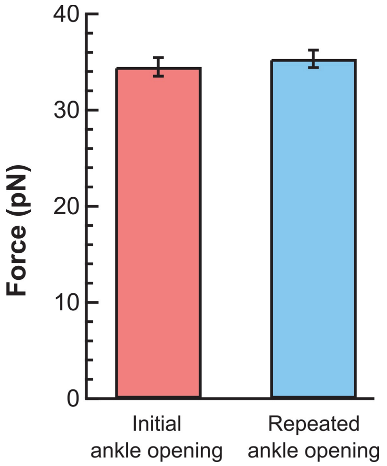

Figure S11. Comparison between initial and repeated ankle-opening force [34 ± 1 pN (mean ± SEM; N = 40) vs. 35 ± 1 pN (N = 40)] acquired at v = 1000 nm/s. For the initial ankle-opening data, a single force-extension curve was acquired at 40 distinct surface locations completely detaching the polyprotein from the tip. For the repeated ankle opening, the data were acquired from two spots with the constant-velocity pulling protocol that also fully stretched the polyprotein and detached from the tip followed by an immediate return of the cantilever to the surface and the process was then restarted. This process had a short dwell (t < 100 ms) for tip attachment as described in the methods section of the main text. The main difference in this revised pulling protocol was that the data was repeatedly acquired from the same spot with the initial ankle-opening force from each spot excluded from the analysis. Data for both the conditions were acquired with the same individual cantilever for improved precision.[13,14] All 40 repeated forces-extension curves showed the expected ankle-opening mechanical fingerprint despite the brief dwell at the surface.

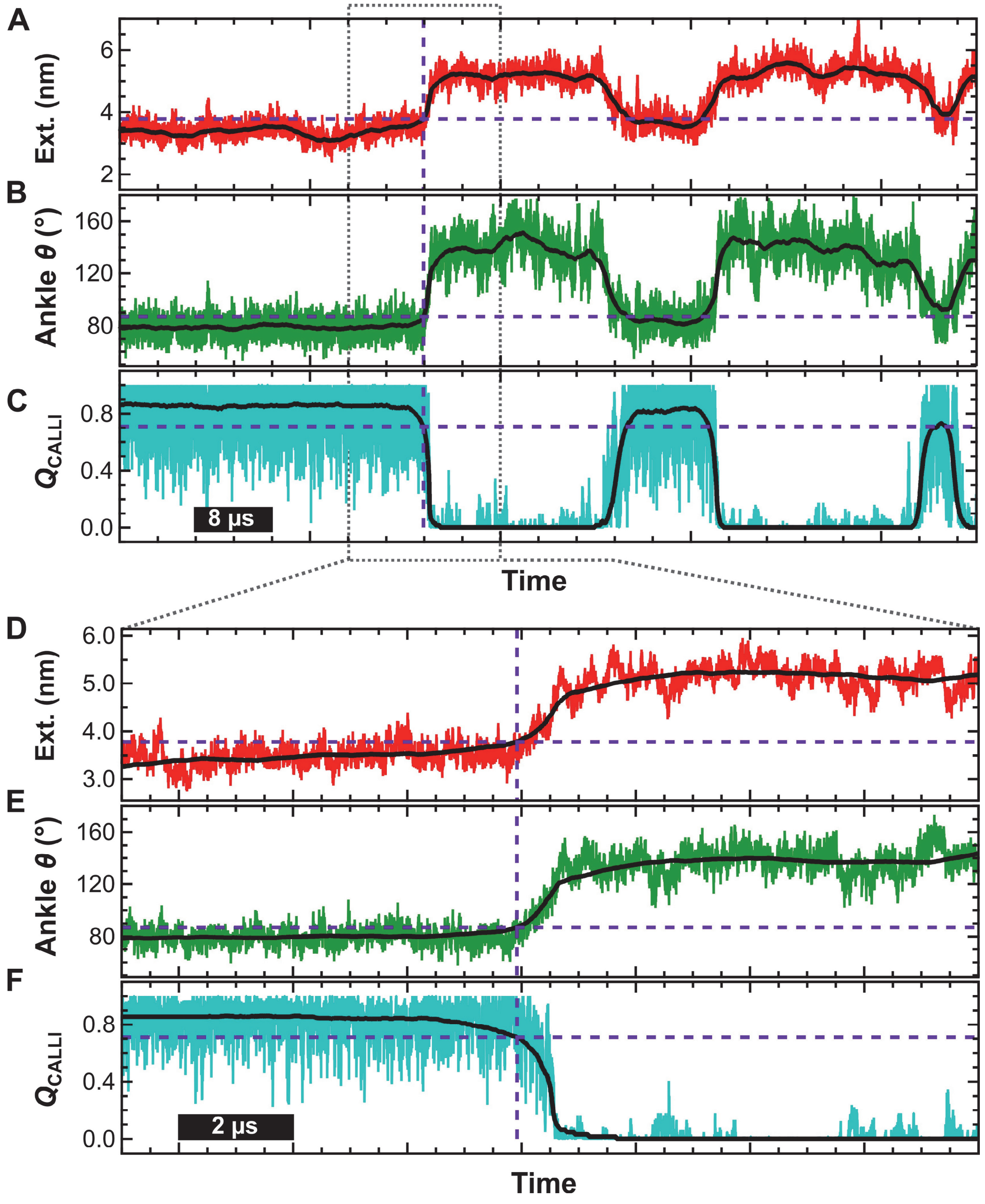

Figure S12. Deducing angular rotation to transition state using the coarse-grained simulation. (**A**) Extension plotted as a function of time (red) for a trajectory segment undergoing transitions between N and O states where the horizontal dashed line represents the increase in extension corresponding to the experimentally obtained distance to transition state ($\Delta x^{\ddagger} = 0.58$ nm). Vertical dashed line represents the time when horizontal dashed line intersects the smoothed extension (black). (**B**) Computationally obtained angle between H_1 and H_2 in LA^{RLC} plotted as a function of time. The horizontal dashed line represents the angle between H_1 and H_2 at the time when the extension matches the transition state location. (**C**) Fraction of native contacts in CALLI (Q_{CALLI}) plotted as a function of time. Horizontal dashed line represents Q_{CALLI} at the transition state. (**D–F**) A higher time resolution depiction of panels A–C

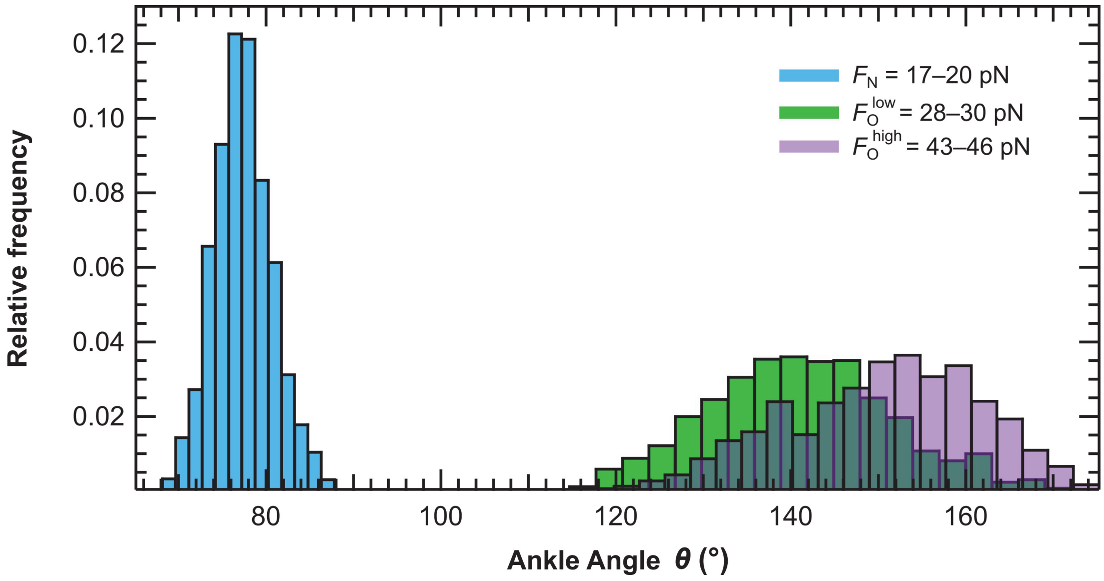

Figure S13. Histogram of ankle angles under force in the native and open state obtained from 17.6-µs trajectory segments smoothed over a 100-ns window using a second-order Savitzky–Golay filter. The distribution of angles in the native state (blue) is narrower [77 ± 3° (mean ± SD) @ 17–20 pN)] than the fluctuations of the ankle angle in open state [140 ± 11° @ 28–30 and 149 ± 11° @ 43–46 pN). Note, the large fluctuations and the shift to larger ankle angles at higher forces show the open-ankle state is not itself rigid, rather it is consistent with being described as a compliant, extensible hinge.

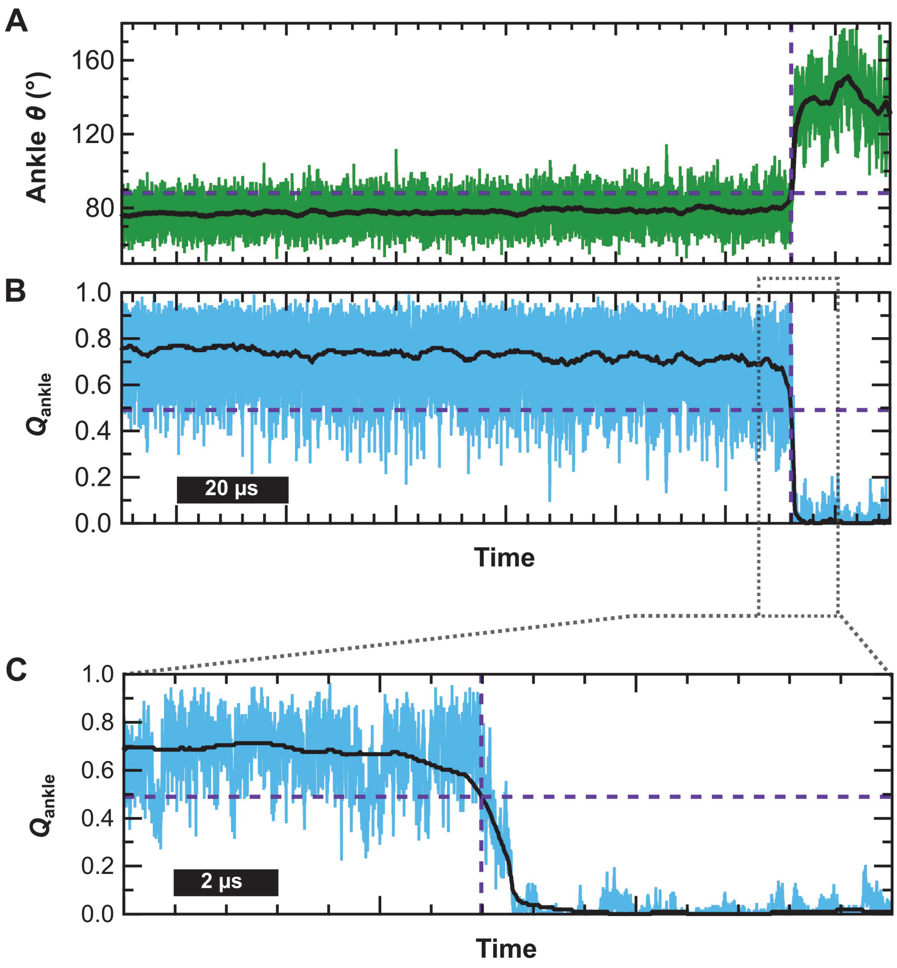

Figure S14. Deducing the fraction of persistent native contacts (Q_{ankle}) that stabilizes the native state against ankle opening at the predicted transition state. (**A**) Computationally deduced ankle angle between the two helices H_1 and H_2 plotted as a function of time (green) for a trajectory segment undergoing an N→O transition, where the horizontal dashed line represents the average ankle angle at the transition state (θ = 88°) and the vertical line represents the time at which θ = 88°. (**B**) The fraction of persistent native contacts that stabilize the native state against ankle opening plotted as a function of time. The vertical line represents the time at which the ankle angle matches its average value at the transition state and the horizontal line represents its value at that time (Q = 0.49). (**C**) A higher time-resolution plot shows the trajectory near the transition.

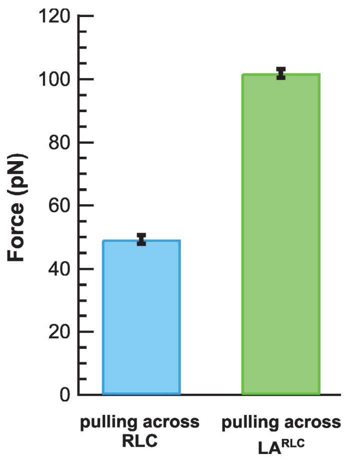

Figure S15. A bar graph shows simulated unfolding force for the EF_c domain when pulling across RLC [49 ± 1 pN (mean ± SEM), $N = 5$] and its mechanical detachment when pulling across the LA^{RLC} (102 ± 1 pN; $N = 27$).

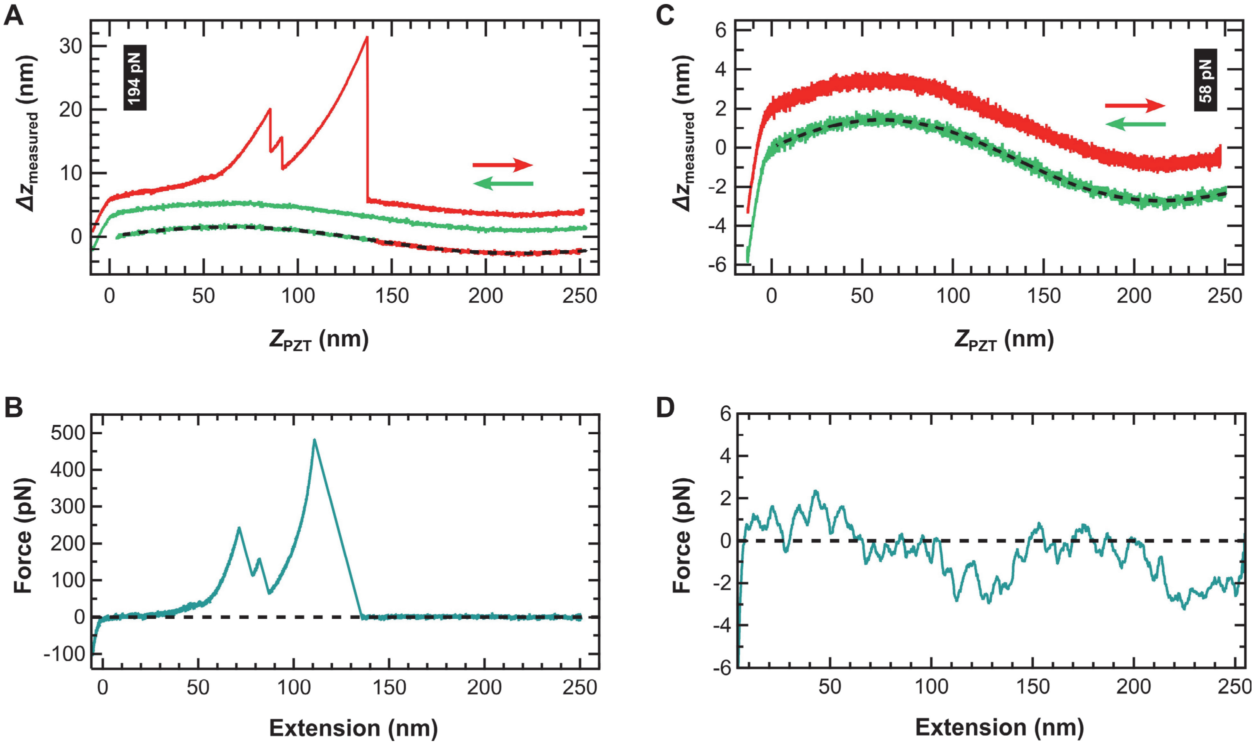

Figure S16. Procedure depicting removal of optical-interference artifact (OIA). (A) Measured cantilever deflection $\Delta z_{\mathrm{Measured}}$ vs. AFM stage movement (Z_{PZT}). The approach (green) and retract (red) curves show an interference-fringe-like pattern. Traces offset vertically for clarity and scale bar represents the force for the corresponding $\Delta z_{\mathrm{Measured}}$. The retract curve after protein detachment was concatenated with part of the approach curve and fitted with Eq. 1 (dashed line). (**B**) Force-extension curve after removal of the OIA. (**C**) $\Delta z_{\mathrm{Measured}}$ for an approach and retract cycle without protein attachment. (**D**) Corrected force-extension curve after removal of the OIA from a retraction curve not attached to a molecule illustrates the force residuals for this process (data smoothed to 100 Hz). Deviations from zero force are below 2 pN in the region where the RLC complex unfolds (*i.e.*, extension = 0–100 nm).

Table S1. HCM-causing mutations in LA^{RLC} from Parker & Peckham.[8]

Mutation
L811P
Q815P
N817K
R819Q
A820D
F821S
M822L, M822V, M822T
G823E
V824A, V824L, V824I
W827C
P828S
Y833C, Y833H
F834L, F834Y
K835T
I836M, I836T
P838L
L840M
S842R, S842N, S842G
E844K
R845G
E846Q, E846K

Table S2. (**A**) Sequence logos showing amino acid conservation for the lever arm derived from the alignment of human myosin isoform MYH1, MYH2, MYH3, MYH4, MYH6, MYH7, MYH7b, MYH8, and MYH13. (**B**) Sequence logos derived from the alignment of MYH7 across different species *(Homo sapiens, Rattus norvegicus, Mus musculus, Canis lupus familiaris, Ailuropoda melanoleuca, Bos Taurus, Oryctolaguscuniculus, Equus caballus, Sus Scrofa, Pongo Abelli)*. Residues are colored using a gradient from blue (hydrophilic) to red (hydrophobic). The height of each column reflects the conservation at that position.

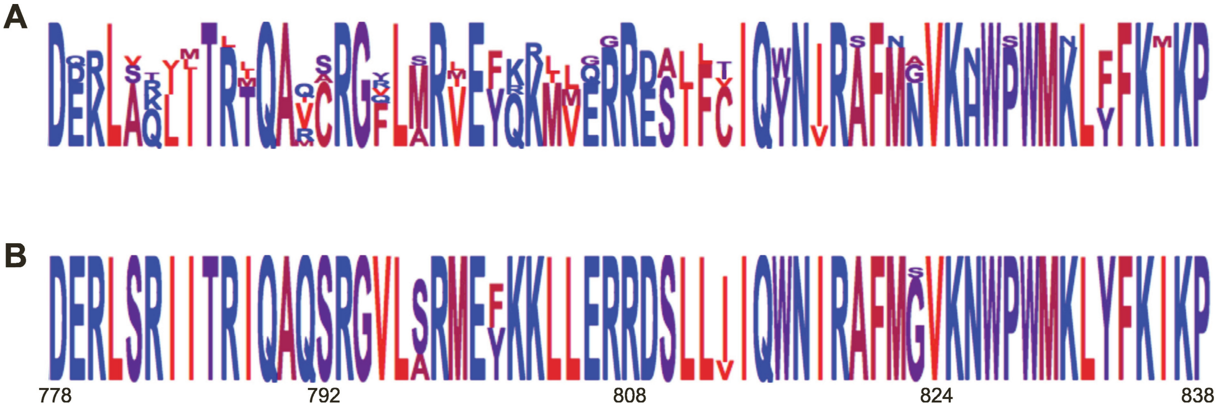

Table S3. The slightly extended amino-acid sequence used for the LA^{RLC} ranging from residues 810–846, where the extra amino acids were added due to sub-stoichiometric binding of the RLC to human β-cardiac myosin S1 ending in the absence of those added amino acids (Ruppel, K.M. and Spudich, J.A. Stanford University, Stanford, CA, Personal communication, 2025).

SLLVIQWNIRAFMGVKNWPWMKLYFKIKPLLKSAERE

810 846

Table S4. A list of the identified coarse-grained interactions beyond nearest-neighbor interactions where SC refers to the side chain of the designated amino acid in the RLC and C_α refers to an interaction with the "bead" in the simulation located at the location of the amino acid's alpha carbon. In this simulation, the side chain beads have the chemical properties associated with their particular amino acid.[15]

Res ID 1	Res ID 2	Res name 1	Res name 2
20_{SC}	90_α	SC-MET	C_α-LEU
20_α	91_α	C_α -MET	C_α-LYS
20_{SC}	91_α	SC-MET	C_α-LYS
20_α	92_{SC}	C_α-MET	SC-GLY
20_{SC}	92_{SC}	SC-MET	SC-GLY
20_α	92_α	C_α-MET	C_α-GLY
20_{SC}	92_α	SC-MET	C_α-GLY
20_α	93_α	C_α-MET	C_α-ALA
21_{SC}	90_{SC}	SC-PHE	SC-LEU
21_{SC}	90_α	SC-PHE	C_α-LEU
21_{SC}	91_α	SC-PHE	C_α-LYS
21_α	92_α	C_α-PHE	C_α-GLY
21_{SC}	92_{SC}	SC-PHE	SC-GLY
21_α	92_{SC}	C_α-PHE	SC-GLY
21_{SC}	92_α	SC-PHE	C_α-GLY
21_{SC}	93_{SC}	SC-PHE	SC-ALA
21_α	93_α	C_α-PHE	C_α-ALA
21_α	93_{SC}	C_α-PHE	SC-ALA
21_{SC}	93_α	SC-PHE	C_α-ALA
21_α	94_α	C_α-PHE	C_α-ASP
21_α	95_{SC}	C_α-PHE	SC-PRO
22_α	94_α	C_α-GLU	C_α-ASP
22_{SC}	94_{SC}	SC-GLU	SC-ASP
22_{SC}	94_α	SC-GLU	C_α-ASP
22_α	95_{SC}	C_α-GLU	SC-PRO
22_α	95_α	C_α-GLU	C_α-PRO
22_{SC}	95_{SC}	SC-GLU	SC-PRO
22_{SC}	95_α	SC-GLU	CAPRO
24_α	95_{SC}	C_α-THR	SC-PRO
24_{SC}	95_{SC}	SC-THR	SC-PRO
24_{SC}	95_α	SC-THR	C_α-PRO
25_{SC}	92_{SC}	SC-GLN	SC-GLY
25_{SC}	92_α	SC-GLN	C_α-GLY
25_{SC}	93_{SC}	SC-GLN	SC-ALA
25_{SC}	93_α	SC-GLN	C_α-ALA
25_{SC}	94_{SC}	SC-GLN	SC-ASP
25_{SC}	94_α	SC-GLN	C_α-ASP
25_{SC}	95_{SC}	SC-GLN	SC-PRO
25_α	95_α	C_α-GLN	C_α-PRO
25_α	95_{SC}	CA-GLN	SC-PRO
25_{SC}	95_α	SC-GLN	C_α-PRO

References:

(1) Proksch, R.; Schaffer, T. E.; Cleveland, J. P.; Callahan, R. C.; Viani, M. B., Finite Optical Spot Size and Position Corrections in Thermal Spring Constant Calibration. *Nanotechnology* **2004**, *15*, 1344–1350.
(2) Yin, J.; Lin, A. J.; Golan, D. E.; Walsh, C. T., Site-Specific Protein Labeling by Sfp Phosphopantetheinyl Transferase. *Nat. Protoc.* **2006**, *1*, 280–285.
(3) Schoeler, C.; Malinowska, K. H.; Bernardi, R. C.; Milles, L. F.; Jobst, M. A.; Durner, E.; Ott, W.; Fried, D. B.; Bayer, E. A.; Schulten, K.; Gaub, H. E.; Nash, M. A., Ultrastable Cellulosome-Adhesion Complex Tightens under Load. *Nat. Commun.* **2014**, *5*, 5635.
(4) Yu, H.; Siewny, M. G. W.; Edwards, D. T.; Sanders, A. W.; Perkins, T. T., Hidden Dynamics in the Unfolding of Individual Bacteriorhodopsin Proteins. *Science* **2017**, *355*, 945–950.
(5) Schoeler, C.; Bernardi, R. C.; Malinowska, K. H.; Durner, E.; Ott, W.; Bayer, E. A.; Schulten, K.; Nash, M. A.; Gaub, H. E., Mapping Mechanical Force Propagation through Biomolecular Complexes. *Nano Lett.* **2015**, *15*, 7370–7376.
(6) Sethi, A.; Eargle, J.; Black, A. A.; Luthey-Schulten, Z., Dynamical Networks in TRNA:Protein Complexes. *Proc Natl Acad Sci U S A* **2009**, *106*, 6620–6625.
(7) Schrödinger, LLC. *The Pymol Molecular Graphics System*, Version 2.6.0a0; Schrödinger, LLC: New York, NY, 2023.
(8) Parker, F.; Peckham, M., Disease Mutations in Striated Muscle Myosins. *Biophys. Rev.* **2020**, *12*, 887–894.
(9) Edwards, D. T.; Faulk, J. K.; LeBlanc, M. A.; Perkins, T. T., Force Spectroscopy with 9-μs Resolution and Sub-pN Stability by Tailoring AFM Cantilever Geometry. *Biophys. J.* **2017**, *113*, 2595–2600.
(10) Grinzato, A.; Auguin, D.; Kikuti, C.; Nandwani, N.; Moussaoui, D.; Pathak, D.; Kandiah, E.; Ruppel, K. M.; Spudich, J. A.; Houdusse, A.; Robert-Paganin, J., Cryo-EM Structure of the Folded-Back State of Human β-Cardiac Myosin. *Nat. Commun.* **2023**, *14*, 3166.
(11) Dietz, H.; Rief, M., Protein Structure by Mechanical Triangulation. *Proc. Natl. Acad. Sci. U.S.A.* **2006**, *103*, 1244–1247.
(12) Cao, Y.; Li, H., Polyprotein of GB1 Is an Ideal Artificial Elastomeric Protein. *Nat. Mater.* **2007**, *6*, 109–114.
(13) Otten, M.; Ott, W.; Jobst, M. A.; Milles, L. F.; Verdorfer, T.; Pippig, D. A.; Nash, M. A.; Gaub, H. E., From Genes to Protein Mechanics on a Chip. *Nat. Methods* **2014**, *11*, 1127–1130.
(14) Pimenta-Lopes, C.; Suay-Corredera, C.; Velázquez-Carreras, D.; Sánchez-Ortiz, D.; Alegre-Cebollada, J., Concurrent Atomic Force Spectroscopy. *Comm. Phys.* **2019**, *2*.
(15) Liu, Z.; Reddy, G.; O'Brien, E. P.; Thirumalai, D., Collapse Kinetics and Chevron Plots from Simulations of Denaturant-Dependent Folding of Globular Proteins. *Proc. Natl. Acad. Sci. U.S.A.* **2011**, *108*, 7787–7792.
